# Phosphoproteomic analysis of the response of maize leaves to drought, heat and their combination stress

**DOI:** 10.3389/fpls.2015.00298

**Published:** 2015-05-05

**Authors:** Xiuli Hu, Liuji Wu, Feiyun Zhao, Dayong Zhang, Nana Li, Guohui Zhu, Chaohao Li, Wei Wang

**Affiliations:** ^1^State Key Laboratory of Wheat and Maize Crop Science, Collaborative Innovation Center of Henan Grain Crops, College of Life Science, Henan Agricultural UniversityZhengzhou, China; ^2^Jiangsu Academy of Agricultural Sciences Institute of BiotechnologyNanjing, China; ^3^Guangdong Provincial Key Laboratory of Protein Function and Regulation in Agricultural Organisms, College of Life Sciences, South China Agricultural UniversityGuangzhou, China

**Keywords:** phosphoproteins, phosphoproteomics, iTRAQ labeling, drought and heat, maize

## Abstract

Drought and heat stress, especially their combination, greatly affect crop production. Many studies have described transcriptome, proteome and phosphoproteome changes in response of plants to drought or heat stress. However, the study about the phosphoproteomic changes in response of crops to the combination stress is scare. To understand the mechanism of maize responses to the drought and heat combination stress, phosphoproteomic analysis was performed on maize leaves by using multiplex iTRAQ-based quantitative proteomic and LC-MS/MS methods. Five-leaf-stage maize was subjected to drought, heat or their combination, and the leaves were collected. Globally, heat, drought and the combined stress significantly changed the phosphorylation levels of 172, 149, and 144 phosphopeptides, respectively. These phosphopeptides corresponded to 282 proteins. Among them, 23 only responded to the combined stress and could not be predicted from their responses to single stressors; 30 and 75 only responded to drought and heat, respectively. Notably, 19 proteins were phosphorylated on different sites in response to the single and combination stresses. Of the seven significantly enriched phosphorylation motifs identified, two were common for all stresses, two were common for heat and the combined stress, and one was specific to the combined stress. The signaling pathways in which the phosphoproteins were involved clearly differed among the three stresses. Functional characterization of the phosphoproteins and the pathways identified here could lead to new targets for the enhancement of crop stress tolerance, which will be particularly important in the face of climate change and the increasing prevalence of abiotic stressors.

## Introduction

In the past decades, progress on increasing crop yields using semi-empiric breeding and genetics has reached a plateau, which is largely linked to increasingly adverse environmental conditions, especially drought and heat stress (Parent and Tardieu, 2014). In the field, it is often the simultaneous occurrence of several abiotic stressors, which are most lethal to crops. Heat, drought and their combination are the severer stressors for crops and are responsible for most of production losses (Lobell et al., 2011; Suzuki et al., 2014). Moreover, global climate change may increase the occurrence and distribution of these stressors, causing a further reduction of productivity (Rasul et al., 2011).

Recent studies have demonstrated that plant responses to the combinations of two or more stressors are unique and cannot be directly extrapolated from the response to a single stress. In Arabidopsis response to combination stress, some 61% of the transcriptome changes are not predictable from the response to single stress, and plants prioritized between potentially antagonistic responses for only 5–10% of the responding transcripts (Rasmussen et al., 2013). In wheat response to combination of drought and heat stress, proteomic analysis indicates that few common proteins are observed responding to single and multiple high-temperature events (Yang et al., 2011); we obtained similar results in maize (Hu et al., 2010). So, the simultaneous occurrence of several stress results in highly complex responses of plants; extrapolated the response to combined stresses is largely controlled by different, and sometimes opposing, signaling pathways that may interact with and inhibit each other (Vile et al., 2012; Suzuki et al., 2014).

Plants respond to stress with a wide range of modifications that cause to changes at the morphological, cellular, physiological, biochemical, and molecular levels (Lopes and Reynolds, 2011; Aprile et al., 2013). Overall, protein phosphorylation plays a critical role in regulating many biological functions, including stress responses by signal transduction. Phosphorylation and dephosphorylation can switch many regulatory proteins and enzymes on and off, thus control a wide range of cellular processes and signal relays (Yang et al., 2010). In recent years, global quantitative analysis of protein expression and phosphorylation has been performed using iTRAQ-based quantitative proteomic and LC-MS/MS methods (Alvarez et al., 2014; Han et al., 2014), which facilitate the simultaneous detection of changes in protein expression and phosphorylation levels under control and stressed conditions.

Large-scale phosphoproteomic analyses have been conducted on crops, especially crops response to stress. In wheat seedlings leaves response to drought stress, some phosphoproteins related to drought tolerance and osmotic regulation exhibit significant phosphorylation changes; there are commonalities and differences of phosphoproteins in different cultivars of bread wheat under drought stress (Lv et al., 2014; Zhang et al., 2014). In maize leaves response to drought stress, a total of 138 phosphopeptides display highly significant changes and most phosphorylation changes do not reflect protein abundance variation. These proteins influenced epigenetic control, gene expression, cell cycle-dependent processes and phytohormone-mediated responses. Bonhomme et al. (2012). These results provide a series of phosphoproteins and phosphorylation sites and a potential network of phosphorylation signaling cascades in wheat seedling leaves. However, little information is available regarding the changes of phosphoproteins and the phosphorylation sites of many stress-responsive protein kinases under combined drought and heat stress. Therefore, characterizing posttranslational modifications of these proteins in crops response to combined stress are very important for understanding the associated signaling pathways and mechanisms for tolerance to stress.

In many regions of the world, maize is an important cereal crop grown mainly in semi-arid environments that are characterized by water scarcity and high temperature, two conditions that usually occur simultaneously in the field. Exposing plants to a combined stress may lead to agonistic or antagonistic responses or cause to some responses that are potentially unrelated to those response to the corresponding single stress. So, to analyze such responses, we performed iTRAQ-based phosphoproteomic analyses in maize exposed to heat, drought and their combination. Furthermore, bioinformatics analyses were conducted to confirm the constitutive nature of the different stress-responsive phosphorylated proteins. This work would provide a basis for further elucidating maize endurance to drought, heat and their combination stress.

## Materials and methods

### Plant material and stress treatments

Maize seeds (Zhengdan 958) were used in the experiments. Zhengdan 958 is a high-yield maize hybrid that is grown in China. The seeds were surface-sterilized for 10 min in 2% hypochlorite, washed in distilled water and germinated on moistened filter paper. Maize seedlings were grown in Hoagland's nutrient solution in a light chamber under 400 μmol m^−2^ s^−1^ photosynthetically active radiation, a 14/10-h day/night cycle, a day/night temperature of 28/22°C, and a relative humidity of 75%. When the fifth leaves were fully expanded, the seedlings were subjected to various treatments.

Drought stress was imposed by placing the seedlings in PEG solution (−0.7 MPa) for 8 h at 28°C and 40% relative humidity. Heat stress was applied by raising the temperature from 28 to 42°C at an interval of 2°C/h and then kept at 42°C for 1 h, for a total of 8 h. Therefore, each stress treatment lasted 8 h. The combined stress consisted of simultaneous treatment with PEG and heat stress. The control seedlings were kept at 28°C and 75% relative humidity. Then, the expanding leaves (the fifth from the bottom) of the treated and untreated seedlings were sampled, immediately frozen in liquid N_2_, and stored at −80°C until analysis. Three biological replicates were performed for each treatment.

### Protein extraction

Total proteins from the fifth newly-expanded leaves of the maize seedlings were extracted according to the method reported by Wang et al. (2013) and Zhang et al. (2014). Approximately 0.5 g of fresh leaves from each biological replicate were ground into a fine power in liquid N_2_ in a mortal and further ground in a 4 ml SDS buffer (30% sucrose, 2% SDS, 100 mM Tris–HCl, pH 8.0, 50 mM EDTA-Na_2_, 20 mM DTT) and 4 ml phenol (Tris-buffered, pH 8.0) in a 10 ml tube, followed by the addition of 1 mM phenylmethanesulfonyl fluoride (PMSF) and PhosSTOP Phosphatase Inhibitor Cocktail (one tablet/10 ml; Roche, Basel, Switzerland) to inhibit protease and phosphatase activity. The mixture was thoroughly vortexed for 30 s and the phenol phase was separated by centrifugation at 14,000 × g and 4°C for 15 min. The upper phenol phase was pipetted into fresh 10 ml tubes and 4-fold volumes of cold methanol plus 100 mM ammonium acetate were added. After centrifugation at 14,000 × g and 4°C for 15 min, the supernatant was carefully discarded and the precipitated proteins were washed twice with cold acetone. Finally, the protein mixtures were harvested by centrifugation. Using a 2-D Quant Kit (Amersham Bioscience, America) containing bovine serum albumin (BSA) (2 mg/ml) as the standard, we carried out the measurement of protein content. To enhance the quantitative accuracy, extracted proteins from every biological replicate were adjusted to the same concentration for the subsequent analysis.

### Protein digestion and iTRAQ labeling

Protein digestion was performed according to the FASP procedure described by Wisniewki et al. (2009) and Lv et al. (2014), and the resulting peptide mixture was labeled using the 4-plex iTRAQ reagent according to the manufacturer's instructions (Applied Biosystems). Briefly, 200 μg of protein from each sample was mixed with 30 μl of STD buffer (4% SDS, 100 mM DTT, 150 mM Tris-HCl pH 8.0). The detergent, DTT and the other low-molecular-weight components were removed using UA buffer (8 M urea, 150 mM Tris-HCl pH 8.0) with repeated ultrafiltration (Microcon units, 30 kD). Then, 100 μl of 0.05 M iodoacetamide in UA buffer was added to block reduced cysteine residues, and the samples were incubated for 20 min in darkness. The filters were washed three times with 100 μl of UA buffer, then twice with 100 μl of DS buffer (50 mM triethylammoniumbicarbonate at pH 8.5). Finally, the protein suspensions were digested with 2 μg of trypsin (Promega) in 40 μl of DS buffer overnight at 37°C, and the resulting peptides were collected as a filtrate. The peptide content was estimated via UV absorption at 280 nm using an extinction coefficient of 1.1 per 0.1% (g/l) solution, which was calculated based on the proportion of tryptophan and tyrosine residues in vertebrate proteins.

For labeling, each iTRAQ reagent was dissolved in 70 μl of ethanol and added to the respective peptide mixture. The samples were called control (under no stress), Drought, Heat and DH (combined drought and heat stress) and were labeled with reagent and vacuum dried.

### Enrichment of phosphorylated peptides using TiO_2_ beads

The labeled peptides were mixed, concentrated using a vacuum concentrator and resuspended in 500 μl of loading buffer (2% glutamic acid/65% ACN/2% TFA). Then, TiO_2_ beads were added and then the sample was agitated for 40 min. The sample was centrifuged for 1 min at 5000 g, yielding the first set of beads. The supernatant from the first centrifugation was mixed with more TiO_2_ beads, which were treated as before, yielding the second set of beads. Both sets of beads were combined and washed three times with 50 μl of wash buffer I (30% ACN/3%TFA) and then three times with 50 μl of wash buffer II (80% ACN/0.3% TFA) to remove the remaining non-adsorbed material. Finally, the phosphopeptides were eluted with 50 μl of elution buffer (40% ACN/15% NH_4_OH), lyophilized and subjected to MS analysis.

### Mass spectrometry

For nanoLC-MS/MS analysis, 5 μl of the phosphopeptide solution was mixed with 15 μl of 0.1% (v/v) trifluoroacetic acid, then 10 μl of the mixture was injected into a Q Exactive MS (Thermo Scientific) equipped with an Easy-nLC (Proxeon Biosystems, now Thermo Scientific). The peptide mixture was loaded onto a C18 reversed phase column (15 cm long, 75 μm inner diameter, RP-C18 3 μm, packed in-house) in buffer A (0.1% formic acid) and separated using a linear gradient of buffer B (80% acetonitrile and 0.1% formic acid) over 240 min at a flow rate of 250 nl/min, which was controlled by IntelliFlow technology. The peptides were eluted with a gradient of 0–60% buffer B from 0 to 200 min, 60 to 100% buffer B from 200 to 216 min, and 100% buffer B from 216 to 240 min.

For MS analysis, the peptides were analyzed in positive ion mode. MS spectra were acquired using a data-dependent, top-10 method by dynamically choosing the most abundant precursor ions from the survey scan (300–1800 m/z) for HCD fragmentation. Determination of the target value was based on predictive automatic gain control (pAGC). The duration of dynamic exclusion was 40.0 s. Survey scans were acquired at a resolution of 70,000 at m/z 200 and the resolution for HCD spectra was set to 17,500 at m/z 200. The normalized collision energy was 27 eV, and the under fill ratio, which specifies the minimum percentage of the target value that is likely to be reached at the maximum fill time, was defined as 0.1%. The instrument was run with peptide recognition mode enabled.

### Data analysis

MS/MS spectra were searched against the Uniprot_Zea_mays database (62977 sequences, downloaded June 14th, 2013) and a decoy database using Mascot 2.2 (Matrix Science), which was embedded in Proteome Discoverer 1.4. For protein identification, the following options were used: peptide mass tolerance = 20 ppm; MS/MS tolerance = 0.1 Da; enzyme = trypsin; missed cleavage = 2; fixed modification: carbamidomethyl (C); iTRAQ4plex (K); iTRAQ4plex (N-term); variable modification: oxidation (M), phosphorylation (S/T/Y). The score threshold for peptide identification was set at a 5% false discovery rate (FDR). The PhosphoRS site probability was estimated on the probability (0–100%) of each phosphorylation site being truly phosphorylated. The PhosphoRS site probabilities above 75% indicate that a site is truly phosphorylated.

### Bioinformatics

The molecular functions of the identified proteins were classified according to their gene ontology annotations and their biological functions. The subcellular localization of the unique proteins identified in this study was predicted using the publicly available program WolfPsort (http://wolfpsort.org). Protein-protein interaction networks were analyzed using the publicly available program STRING (http://string-db.org/). STRING is a database of known and predicted protein-protein interactions. The interactions include direct (physical) and indirect (functional) associations, and they are derived from four sources: the genomic context, high-throughput experiments, coexpression and previous knowledge. STRING quantitatively integrates the interaction data from these sources for a large number of organisms and, where applicable, transfers information between these organisms.

Motif-X online software (http://motif-x.med.harvard.edu/motif-x.html) was used to find phosphorylation site motifs in the identified maize proteins and to predict the specificity of these motifs based on the identified phosphopeptide sequences. The parameters were set to peptide length = 21, occurrence = 5, and statistical significance for *p*-values of less than 0.000001.

### Statistical analysis

The phosphoproteins assays were the mean of three replicates. Means were compared by one-way analysis of variance and Duncan's multiple range test at 5% level of significance.

## Results

### Phosphopeptide identification under drought, heat and combined stress

**Figure 1:**
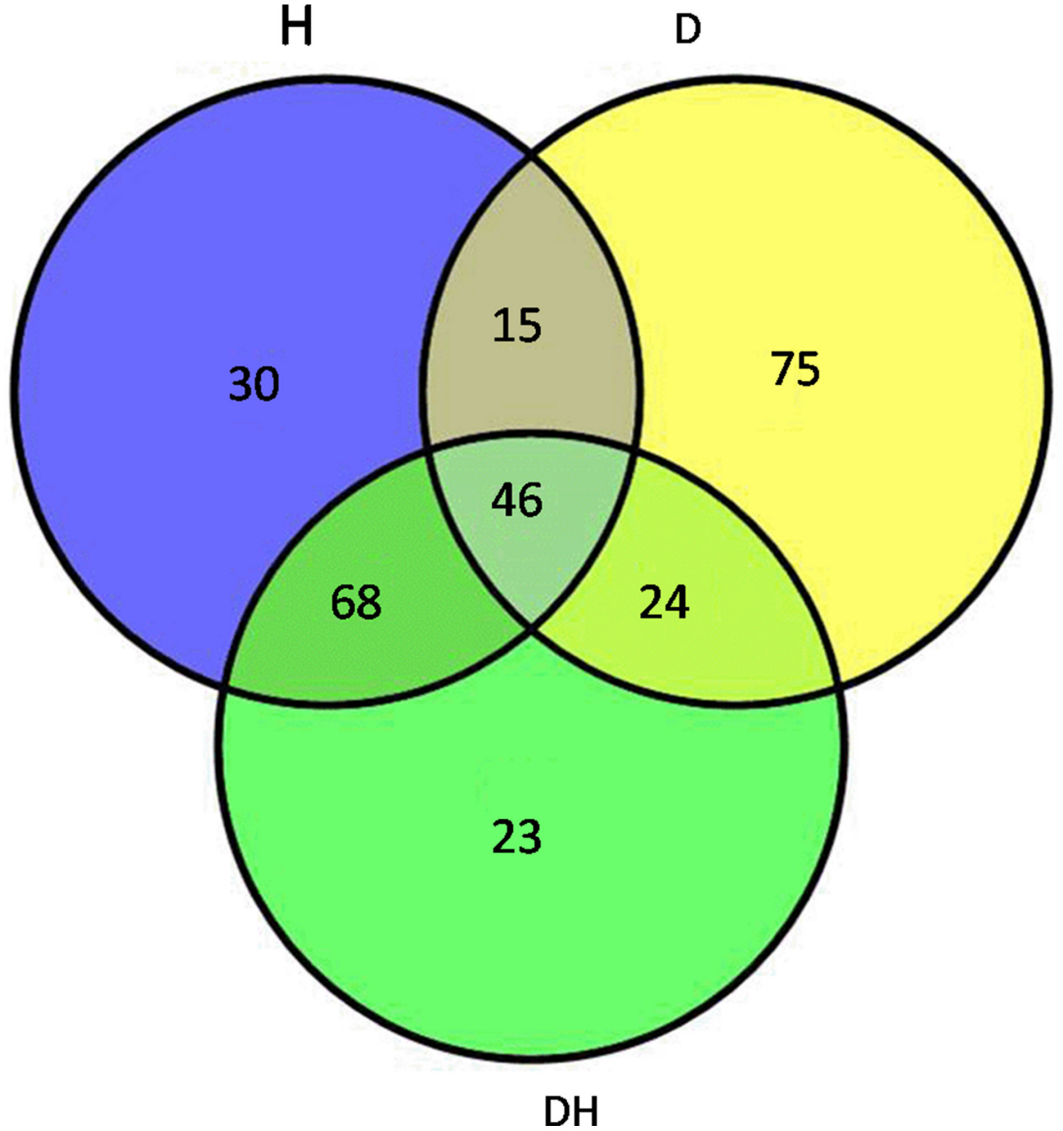
**Venn diagram showing the number of proteins with significant changes in phosphorylation levels in maize leaves exposed to drought (D), heat (H) and combined drought and heat stress (DH)**. The diagram shows the overlap between the results from D, H, and DH.

Maize plants at the five-leaf stage were subjected to drought, heat and their combination stress. Multiplex iTRAQ-based quantitative proteomic and LC-MS/MS methods were performed on the total proteins extracted from the fifth newly-expanded leaves, resulting in the identification of 1367 unique phosphopeptides (Figure 1). These phosphopeptides corresponded to 1039 proteins and contained 2171 non-redundant phosphorylation sites, of which 1313 (60.48%) were serine (S) residues, 649 (29.89%) were threonine (T) residues and 209 (9.63%) were tyrosine (Y) residues at an estimated false discovery rate of 5%. In detail, based on a significant linear regression (*p* < 0.05) and a threshold of ≥1.5-fold or ≤0.66-fold change ratio of stress-induced phosphorylation levels compared with that of the control, the phosphorylation level of 172 phosphopeptides had a significant change under heat, of which 77 were up-regulated and 95 were down-regulated; the phosphorylation level of 149 phosphopeptides had a significant change under drought, of which 69 were up-regulated and 80 were down-regulated; and the phosphorylation level of 144 phosphopeptides had a significant change under the combination stress, of which 70 were up-regulated and 74 were down-regulated. The phosphopeptides with significant changes in phosphorylation levels corresponded to 282 proteins, of which 46 proteins were common under three stress conditions (Table 1), 69 proteins were common under heat stress and the combined stress (Table S1), 24 proteins were common under drought stress and the combined stress (Table S2), 15 proteins were common under drought and heat stress (Table S3), 75 proteins were only identified under drought stress (Table S4), 30 proteins were only identified under heat stress (Table S5), and 23 proteins were only identified under the combined stress (Table S6).

**Table 1:** **The Proteins with significant phosphorylation level changes under D, H, and DH**.

**Protein group accessions**	**Protein name**	**Sequence**	**PhosphoRS site probabilities (only listed >75%)**	**Ratio of phosphorylation level**			***P*-Value**		
				**D/CK**	**H/CK**	**DH/CK**	**D/CK**	**H/CK**	**DH/CK**
B4F7W7	MYB-CC type transfactor	tEPStPAPAsESPtQVGASNR	T(14): 97.1	2.40	2.90	3.62	0.0046	0.0002	0.0001
B4FAB7	CBS domain containing protein	nmESTSPSAASSSSTQLsPR	S(18): 100.0	3.57	2.81	2.43	0.0000	0.0003	0.0090
B4FC96	Zn-containing protein	gsPmPVSSPWsGGALAENNDNIASR	S(2): 99.6	0.59	0.54	0.53	0.0106	0.0318	0.0401
B4FGQ4	Protein kinase superfamily protein	tSSTsDSGR	S(5): 75.6	0.38	0.26	0.50	0.0000	0.0000	0.0259
B4FI93	Zinc finger ccch domain-containing protein 19-like	qNsGNER	S(3): 100.0	2.62	2.88	2.52	0.0019	0.0002	0.0066
B4FKH3	Unknown	kVEcLsGSPPMAISyVsk	S(17): 97.3	0.48	2.49	2.10	0.0004	0.0013	0.0293
B4FKM0	Protochlorophyllide reductase b	aQTAAVsSPSVTPASPSGk	S(7): 81.2	0.471	0.47	0.368	0.0002	0.0072	0.0013
B4FQU2	Translation initiation factor eif-2b beta subunit	tASStGDSDGk	T(5): 94.8	0.63	1.86	4.18	0.0245	0.0266	0.0000
B4FRF2	Oxygen-evolving enhancer protein 1	gGsTGYDNAVALPAGGR	S(3): 96.7	2.30	2.06	1.62	0.0073	0.0104	0.1577
B4FRX1	Splicing arginine serine-rich 7	gGSPTGsksP	S(3): 100.0; S(9): 100.0	1.59	1.90	2.54	0.1348	0.0220	0.0061
B4FUV7	Uncharacterized protein	sLscAER	S(3): 100.0	0.61	0.24	0.19	0.0148	0.0000	0.0000
B4FY17	Phospholipase c	sGSGNIAELPDQGsLR	S(14): 100.0	3.37	2.73	3.35	0.0001	0.0004	0.0004
B4G250	Heat shock protein 3	sIQIsG	S(5): 100.0	0.64	9.30	17.34	0.0303	0.0000	0.0000
B6SRZ6	Ubiquitin-protein ligase	tVsAVGGGGGAPcsPSSR	S(3): 95.3; S(14): 100.0;	2.55	3.33	2.26	0.0025	0.0000	0.0164
B6SS42	Reticulon-like protein b8-like	lFGGGEsR	S(7): 100.0	1.74	1.80	2.46	0.0753	0.0351	0.0079
B6T1H0	tpa: 40s ribosomal protein s9	sEsLAk	S(3): 100.0	2.26	0.16	0.17	0.0084	0.0000	0.0000
B6T1H0	tpa: 40s ribosomal protein s9	aSAAtSA	T(5): 80.0	1.96	0.20	0.26	0.0294	0.0000	0.0000
B6TDN3	Uncharacterized protein LOC100276536	gAsAPPsPAGSPDGLIAALR	S(3): 100.0; S(7): 99.8	4.71	3.77	3.65	0.0000	0.0000	0.0001
B6TH05	Desi-like protein hag1-like	nQASPEsPGNNQNR	S(7): 99.8	5.19	2.84	3.67	0.0000	0.0002	0.0001
B6U114	Probable adp-ribosylation factor gtpase-activating protein agd14-like	tISSASsIGsAEGTSEQTk	T(1): 80.6	1.99	1.66	2.07	0.0259	0.0701	0.0328
B6U0R6	tpa: duf827 domain containing family protein	nEAsVEDLQGmEDVSLSLEEYsELAAk	S(4): 76.0	0.34	0.44	0.43	0.0000	0.0036	0.0070
B6U4K3	Vacuolar-sorting receptor 3-like	sLGLDVk	S(1): 100.0	0.61	0.44	0.54	0.0152	0.0041	0.0457
C0HE61	U2 snrnp-associated surp motif-containing	tsSSSER	S(2): 99.9	4.82	1.95	4.97	0.0000	0.0171	0.0000
C0HIM6	Integrin-linked protein kinase family protein	qLssGAAR	S(3): 100.0; S(4): 100.0	1.71	1.80	1.52	0.0851	0.0351	0.2165
B8A0K2	Calmodulin-binding family protein	tLsGGLQsPR	S(3): 100.0; S(8): 100.0	2.38	1.64	1.97	0.0051	0.0744	0.0465
C0P3W9	Phosphoenolpyruvate carboxykinase	sAPStPKR	S(1): 97.9; S(4): 2.2; T(5): 99.9	0.53	0.23	0.15	0.0019	0.0000	0.0000
C0P3W9	Phosphoenolpyruvate carboxykinase	sAPStPkRsAPTtPIk	S(1): 100.0; S(4): 94.4; T(5): 94.4; T(13): 99.8	0.22	0.22	0.21	0.0000	0.0000	0.0000
C0P3W9	Phosphoenolpyruvate carboxykinase	sAPSTPk	S(1): 100.0	0.58	0.41	0.29	0.0019	0.0000	0.0000
C0P3W9	Phosphoenolpyruvate carboxykinase	gEAAAQGAPstPR	S(10): 100.0; T(11): 100.0	0.64	0.59	0.61	0.0292	0.0664	0.1062
C0PD30	Fructose-bisphosphate aldolase	lAsIGLENTEANR	S(3): 100.0	3.00	3.33	2.67	0.0004	0.0000	0.0039
C4J042	Unknown	ymFAQItcGNGNAPtDSMk	T(7): 90.6	0.50	0.35	0.39	0.0007	0.0002	0.0022
C4J1A8	Probable protein phosphatase 2c 30-like	eQSsPTSNLsPR	S(4): 99.7; S(10): 99.8	12.67	2.23	8.77	0.0000	0.0044	0.0000
C4J4L7	Uncharacterized protein LOC100501611	nsAGALHtSSSIPmSPTAk	T(8): 83.8	2.49	2.78	2.03	0.0032	0.0003	0.0369
C4JBR4	Glycine-rich protein 2	sYGGsWGGGR	S(5): 100.0	2.23	1.76	2.25	0.0097	0.0423	0.0168
F1DJV0	Bzip transcription factor superfamily protein	nNLTEGGAEsDEEIR	S(10): 100.0	2.41	2.34	4.05	0.0044	0.0026	0.0000
K7TNK3	Leucine-rich repeat-containing protein ddb_g0290503-like	qItLSEIk	T(3): 100.0	1.60	1.94	2.20	0.1332	0.0181	0.0201
K7TZQ1	Serine threonine-protein kinase wnk4-like	qYPAsAGsSPSR	S(5): 93.4	2.71	2.20	3.81	0.0013	0.0050	0.0001
K7U162	RNA polymerase ii-associated factor 1 homolog	vEDIDQySEEYsE	S(12): 96.7	0.24	0.44	0.37	0.0000	0.0041	0.0013
K7U573	Nucleic acid binding protein	nQsNVNR	S(3): 100.0	2.70	2.20	2.82	0.0013	0.0052	0.0022
K7U5U1	Hypothetical protein ZEAMMB73_848936	nAPsLGmGMMIk	S(4): 100.0	2.21	2.43	1.71	0.0106	0.0017	0.1138
K7UF98	Protein kinase superfamily protein	gAVAPAVDPsSPSSR	S(10): 78.0	2.25	1.51	1.78	0.0089	0.1336	0.0914
K7V0H0	Probable -trehalose-phosphate synthase	vmsVASPASPTsPSPPAPPR	S(3): 88.1	1.96	1.87	1.52	0.0304	0.0255	0.2158
K7UKJ5	Splicing arginine serine-rich factor 7	sPSYNR	S(1): 100.0	2.11	3.50	4.67	0.0161	0.0000	0.0000
K7VBI0	Hypothetical protein	sIsETTLER	S(3): 100.0	3.40	0.49	1.82	0.0001	0.0129	0.0778
K7VBA5	hypothetical protein	aNsGGQPQAR	S(3): 100.0	1.77	3.12	3.91	0.0666	0.0001	0.0001
Q8W149	Cell division cycle 5-like	eIQTPNPmATPLAsPGPGItPR	S(14): 94.0; T(20): 94.0	5.15	2.94	3.58	0.0000	0.0001	0.0002
Q9LLI8	Probable cellulose synthase a catalytic subunit 2	lTSGQQISGEIPDAsPDR	S(15): 99.9	0.30	0.41	0.31	0.0000	0.0016	0.0002

*CK, control; D, drought stress; H, heat stress; DH, combined drought and heat stress. Each ratio was the average of three replicates*.

### Heat shock proteins

In this study, the phosphorylation levels of seven heat shock proteins (HSPs), including five small HSPs (sHSPs) and two HSP70s, changed significantly under drought, heat or combined both stresses. The phosphorylation levels of three sHSPs (B4G250, BF976 B6T649) were greatly up-regulated under heat and the combined stress. However, under drought, the phosphorylation levels of the three sHSPs did not change significantly. It was worth mentioning that the two phosphopeptides of B4G250 differed significantly in phosphorylation level. In contrast to the three sHSPs, the phosphorylation level of the sHSP C0P2N6 was down-regulated by heat and combined stresses but was not significantly affected by drought stress; the phosphorylation level of sHSP B4FR07 was down-regulated by drought stress. Regarding the two HSP70s, the phosphorylation level of B6SZ69 was significantly up-regulated by drought stress and down-regulated by heat stress but had no significant change under the combined stress. The phosphorylation level of K7WBH2 was significantly up-regulated by drought stress, down-regulated by heat stress and had no significant change under the combined stress.

### Responses of kinases and phosphatases to the three stresses

It was also worth mentioning the responses of enzymes, including kinases and phosphatases, to stresses. Under heat (Table 2), 31 phosphopeptides, including 24 different enzymes, were identified, of which five were kinases and three were phosphatases. Under drought stress (Table 2), 29 phosphopeptides, including 22 unique enzymes, were identified, of which seven were kinases and five were phosphatases. Under the combined stress (Table 2), 29 phosphopeptides corresponding to 21 unique proteins, including five kinases and five phosphatases, were identified. In addition, fructose-bisphosphate aldolase, phosphoenolpyruvate carboxykinase, phospholipase c, probable cellulose synthase catalytic subunit 2, probable protein phosphatase 2c 30-like protein, protochlorophyllide reductase b, serine-threonine protein kinase WNK4-like protein, and ubiquitin-protein ligase had significant change of phosphorylation level under the three stresses; 2-aminoethanethiol dioxygenase-like protein, calcium-dependent protein kinase, ferric-chelate reductase 1-like protein, geranylgeranyl pyrophosphate synthase 4, glycerol 3-phosphate permease, lipid phosphate phosphatase 3 and ubiquitin carboxyl-terminal hydrolase 6-like protein changed significantly in phosphorylation level under drought; and histone-lysine N-methyltransferase family protein, plasma membrane H^+^-transporting ATPase-like protein, and pyruvate orthophosphate dikinase changed significantly in phosphorylation level under the combined stress (Table 2).

**Table 2:**
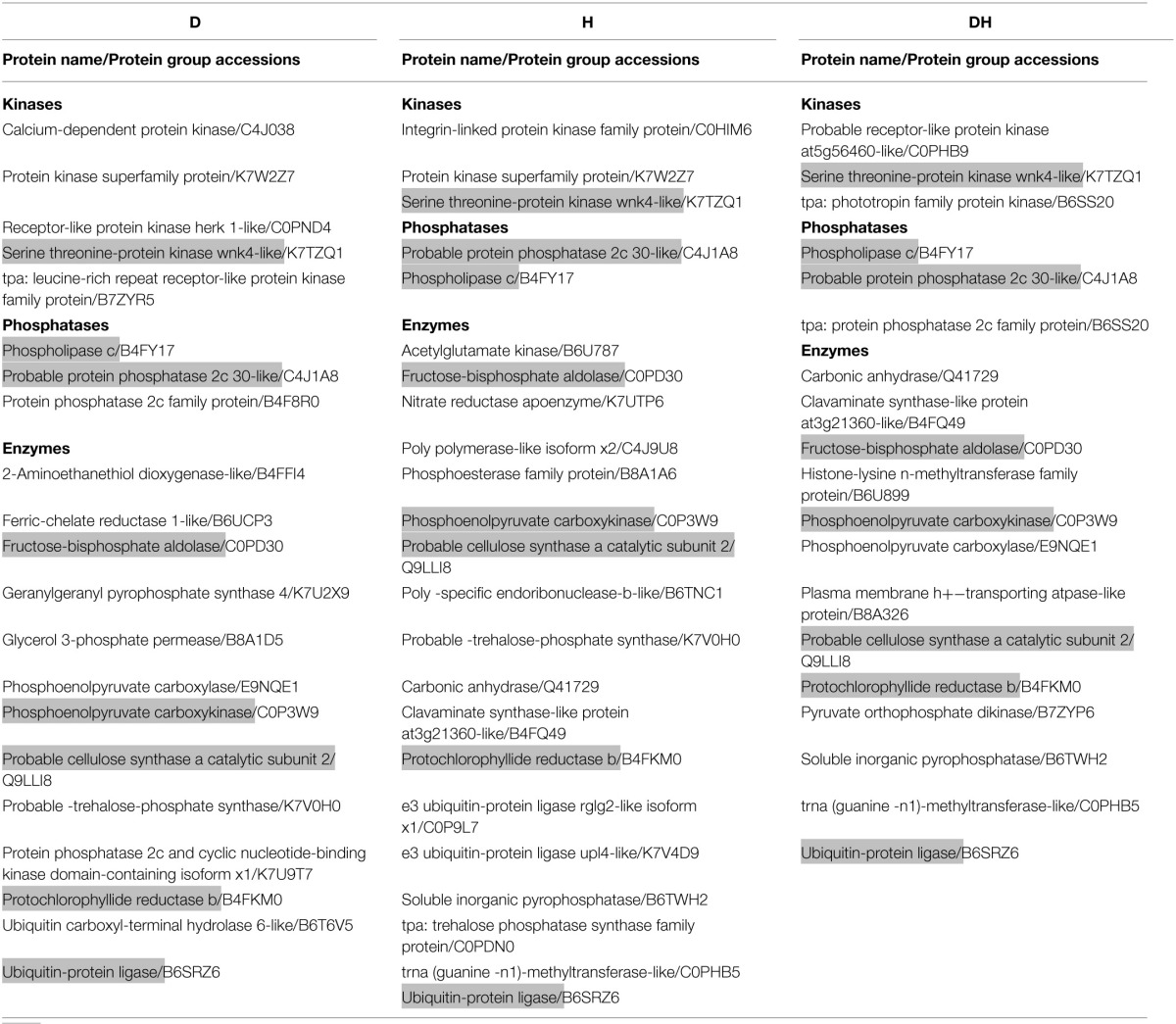
**Identification of kinases, phosphatases and other enzymes under D, H, and DH respectively**.

*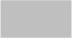 Indicates that these enzymes were common three stress treatments*.

**Figure 2:**
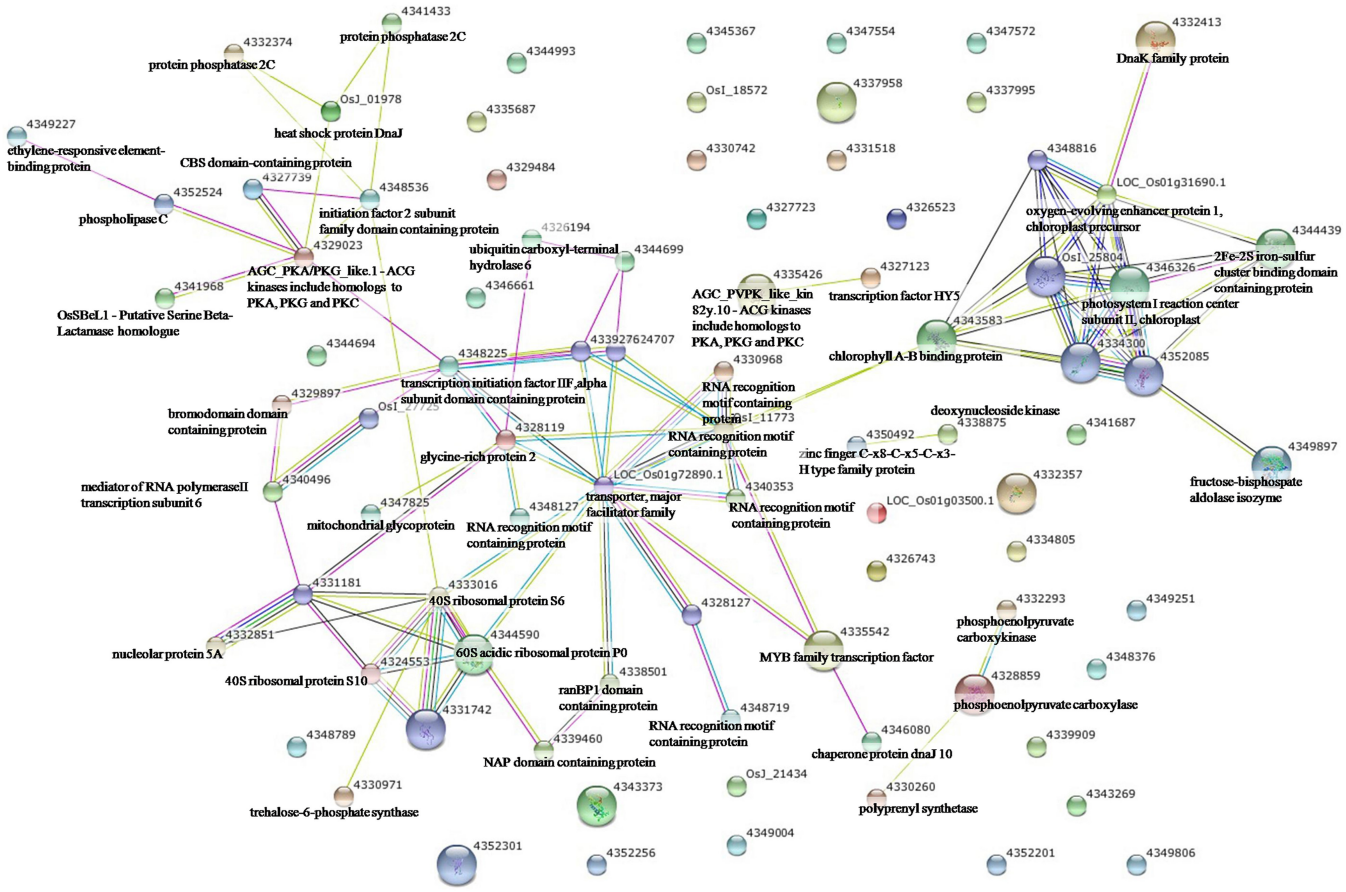
**The protein-protein interaction network analysis among significantly changed proteins in maize seedlings under drought using String software**.

**Figure 3:**
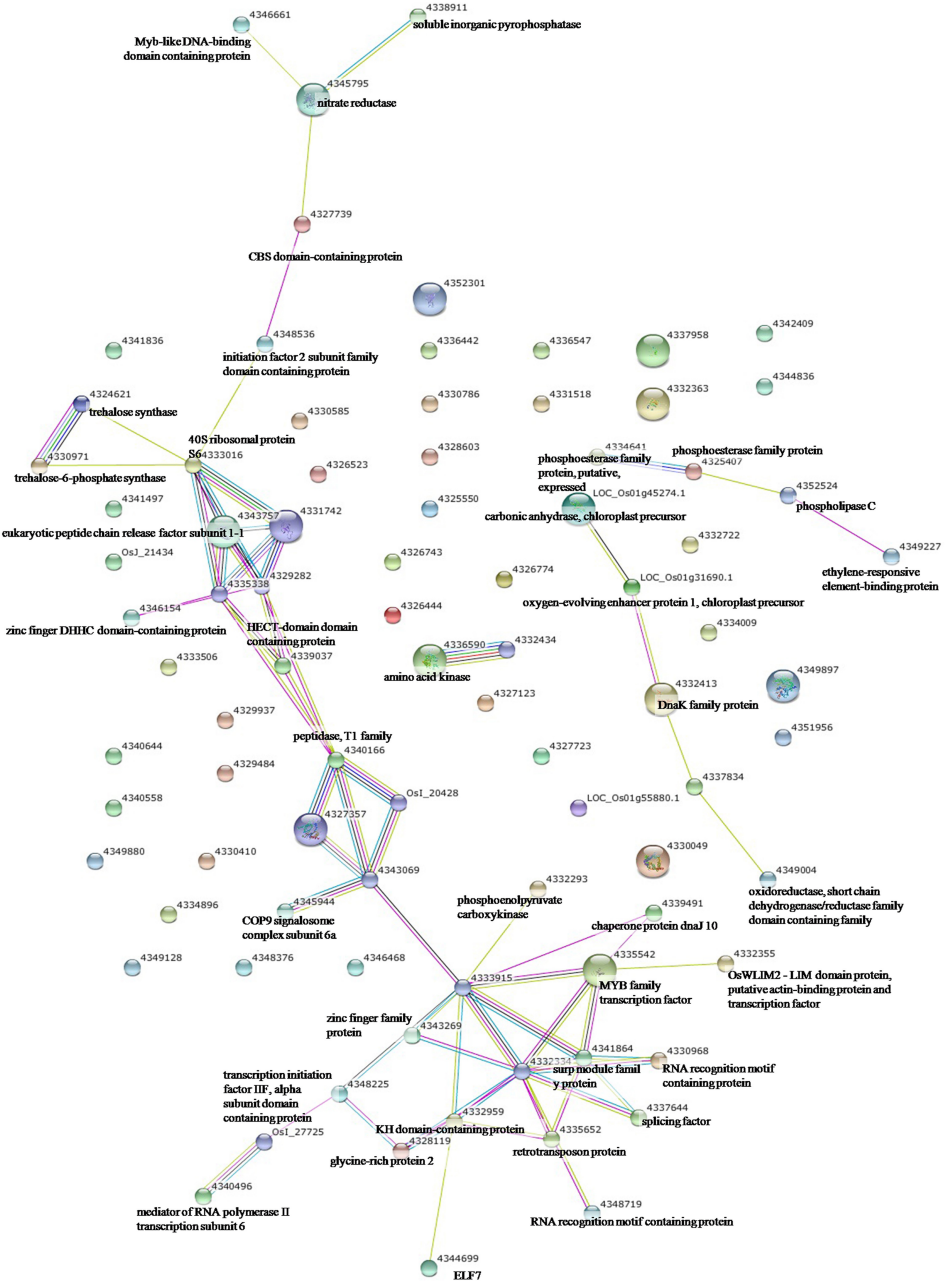
**The protein-protein interaction network analysis among significantly changed proteins in maize seedlings under heat stress using String software**.

**Figure 4:**
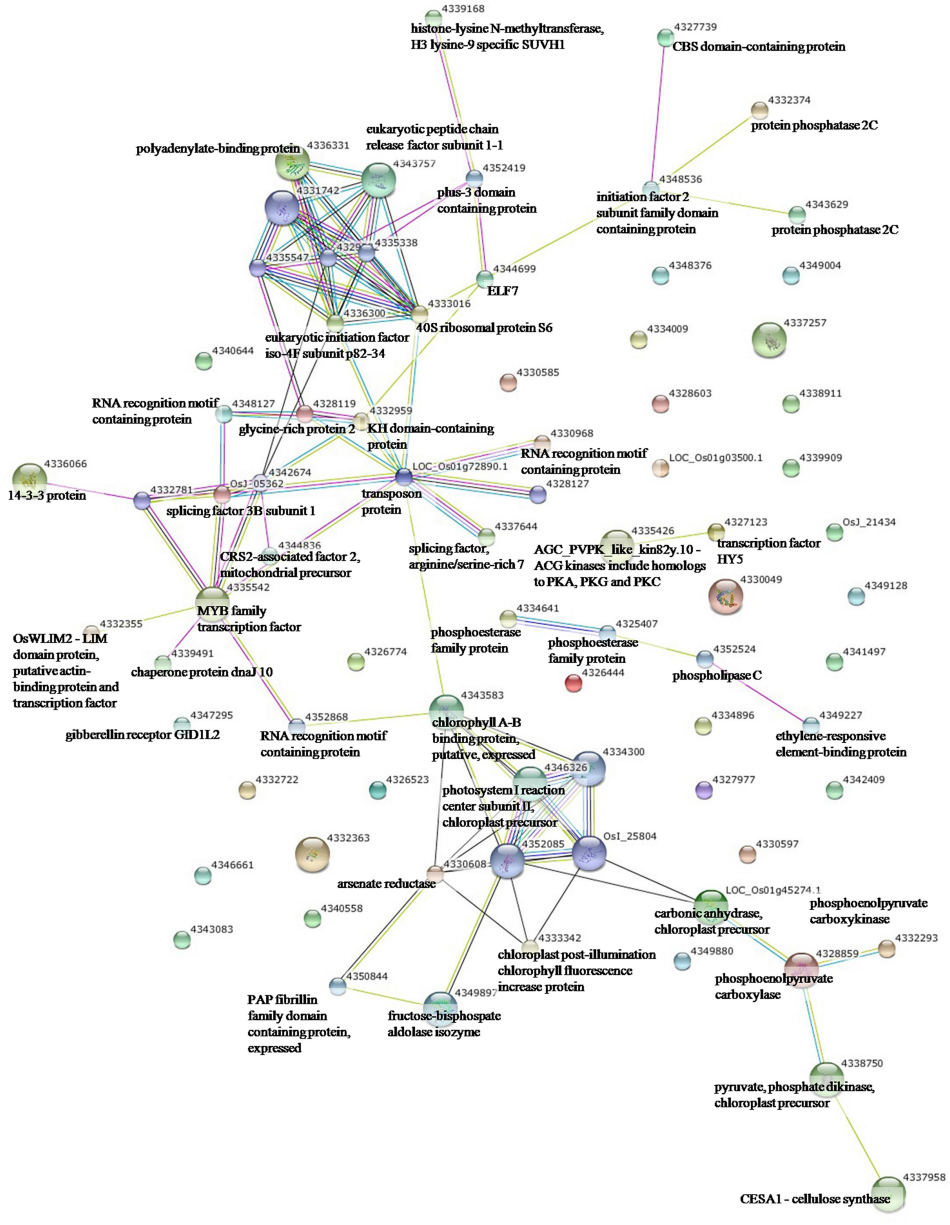
**The protein-protein interaction network analysis among significantly changed proteins in maize seedlings under combined drought and heat stress using String software**.

To elucidate the interactions of the protein kinases/phosphatases and HSPs with other proteins under the three stress treatments, protein-protein interaction analysis of significantly changed phosphoproteins was conducted using STRING software (Figures 2–4). Under drought and the combined stress, AGC_PKAPKG_like.1-ACG kinases (4335426, including homologs of PKA, PKG and PKC) had interactions with transcription factor HY5 (4327123). Protein phosphatase 2C (4341433, 4332374) showed an interactions with initiation factor 2 subunit family domain containing protein (4348536). Under drought, AGC_PKAPKG_like.1-ACG kinases also had interactions with heat shock protein DnaJ (OsJ_01978), initiation factor 2 subunit family domain containing protein (4348536), CBS domain-containing protein (43327739), phospholipase C (4352524) and transcription initiation factor IIF (4348228); protein phosphatase 2C also showed an interaction with heat shock protein DnaJ (OsJ_01978). Under drought and heat stress, a dnaK family protein (HSP70, 4332413) exhibited an interaction with oxygen-evolving enhancer protein 1 (LOC_Os01g31690.1). Under three stress treatments, phospholipase C (4352524) exhibited an interaction with an ethylene-responsive element-binding protein (4349277), and chaperone protein dnaJ 10 (sHSP40, 4346080) interacted with a MYB family transcription factor (4335542). These findings indicated that the AGC_PKA/PKG_like.1-ACG kinases, protein phosphatase 2C and phospholipase C may play an important role in protein substrate phosphorylation/dephosphorylation and that HSPs may play an important protective role as chaperone proteins under drought stress, heat stress and combined both stresses (maize protein query sequences corresponded to rice protein query sequences; see Supplementary Table S7 for drought stress, Table S8 for heat stress, and Table S9 for the combined stress).

### The characteristics of the different phosphorylation states of one protein in response to three stresses

In this study, 19 phosphoproteins (Table 3) were found to have different phosphorylated states. Importantly, these peptides had specific phosphorylation characteristics in response to drought, heat and the combination of both stresses. In particular, phosphoenolpyruvate carboxykinase (C0P3W9) had 11 different phosphorylation sites, and the phosphorylation of sAPStPkR, sAPSTPk, sAPStPkRsAPTtPIk, and gEAAAQGAPstPR changed significantly under the three stresses; sAPttPIk, sAPTTPIk, gEAAAQGAPStPR, and eVDYADNsVTENTR changed significantly under heat and the combined stress conditions; rsAPTtPIk and sAPStPkR changed significantly under drought and the combined drought and heat stress conditions; and gGAHsPFAVAISEEER changed significantly only in response to drought stress. The phosphorylation sites of the other 18 proteins were similar to these. This result shows the diversity of the phosphorylation sites and their specificity in response to different stress treatments.

**Table 3:** **Characteristic of several phosphopeptides belonging to one protein in response to D, H, and DH**.

**Protein group accessions**	**Protein name**	**Sequence**	**Significant changes of phosphorylation level**		
			**D**	**H**	**DH**
A3KLI0	RAB17 protein	sGsSSSSSSEDDGmGGR	9.44 (Y)	–	10.54 (Y)
		sGsSSSSSSEDDGMGGR	1.90 (Y)	1.30 (N)	1.92 (Y)
B4FJG1	Chlorophyll a-b binding protein chloroplastic-like	nEAGGIIGtRFESSDVk	2.64 (Y)	0.71 (N)	1.21 (N)
		nEAGGIIGtR	2.26 (Y)	0.73 (N)	1.34 (N)
B4FRX1	Splicing arginine serine-rich 7	gGSPTGsksP	1.59 (Y)	2.90 (Y)	2.54 (Y)
		sRSPQDQVmsPPPk	0.65 (Y)	1.03 (N)	0.70 (N)
B4FUV7	Uncharacterized protein LOC100275458	sLscAER	0.61 (Y)	0.24 (Y)	0.19 (Y)
		sGsVTNWTSANR	0.82 (N)	0.33 (Y)	0.34 (Y)
		sVSSWSTPPAPPPVQR	0.92 (N)	0.62 (Y)	0.54 (Y)
B4FQK5	Eukaryotic peptide chain release factor subunit 1-1	sFDELsDDDDVYEDsD	0.80 (N)	0.56 (Y)	0.79 (N)
		sFDELsDDDDVYEDSD	0.80 (N)	–	0.54 (Y)
B4FXM8	Nuclear-interacting partner of alk	dTELDEASNETQPEtGSPLRk	0.53 (Y)	0.83 (N)	0.72 (N)
		dTELDEASNETQPETGsPLR	2.06 (Y)	0.90 (N)	0.84 (N)
B4G250	Heat shock protein 3	sIQIsG	0.64 (Y)	9.3 (Y)	17.34 (Y)
		tSsETAAFAGAR	0.90 (N)	4.39 (Y)	8.52 (Y)
B6T1H0	tpa: 40s ribosomal protein s9	sEsLAk	2.26 (Y)	0.16 (Y)	0.17 (Y)
		rSEsLAk	1.33 (N)	0.53 (Y)	0.49 (Y)
		asAAtSA	2.35 (Y)	0.31 (Y)	0.93 (N)
B6SKI1	Photosystem i reaction center subunit ii	gFVAPQLDPSTPSPIFGGStGGLLR	0.63 (Y)	0.80 (N)	0.55 (N)
		atAGEAVTEEAPk	1.35 (N)	1.69 (N)	2.01 (Y)
B6SXN6	Bel1-type homeodomain protein	gAsASNPNNNPGNk	1.11 (N)	0.37 (Y)	0.37 (Y)
		gGASSsGAAAQsPSSAPNkEPPQLSPADR	2.18 (Y)	0.74 (N)	0.78 (N)
B7ZYS1	tpa: phototropic-responsive nph4 family protein	eAPTSSFYGGEsPTPAPSLQGR	0.68 (Y)	0.41 (Y)	0.49 (Y)
		eAPTSSFYGGEsPTPAPsLQGR	1.00 (N)	0.28 (Y)	0.53 (Y)
C0P3W9	Phosphoenolpyruvate carboxykinase	sAPStPkR	2.04 (Y)	1.38 (N)	1.71 (Y)
		sAPStPkRsAPTtPIk	0.22 (Y)	0.22 (Y)	0.21 (Y)
		sAPSTPk	0.58 (Y)	0.41 (Y)	0.29 (Y)
		gEAAAQGAPstPR	0.64 (Y)	0.59 (Y)	0.61 (Y)
		sAPttPIk	0.70 (N)	0.35 (Y)	0.22 (Y)
		sAPTTPIk	1.12 (N)	0.49 (Y)	0.49 (Y)
		gEAAAQGAPStPR	0.90 (N)	0.47 (Y)	0.51 (Y)
		eVDYADNsVTENTR	—	4.83 (Y)	3.43 (Y)
		rsAPTtPIk	0.66 (Y)	0.67 (N)	0.51 (Y)
		sAPStPKR	0.53 (Y)	0.23 (Y)	0.15 (Y)
		gGAHsPFAVAISEEER	0.64 (Y)	0.87 (N)	0.81 (N)
C0P4D8	Dynamin-2b-like isoform 2	qLsIHDNR	1.14 (N)	1.75 (Y)	1.98 (Y)
		aSsPQTDAEQGGGSLk	0.79 (N)	2.02 (Y)	1.85 (Y)
C0P9L7	E3 ubiquitin-protein ligase rglg2-like isoform x1	ssSFSQQSGVYSR	0.88 (N)	0.42 (Y)	0.40 (Y)
		sSsFDQQTSGASQQR	1.34 (N)	0.55 (Y)	0.66 (N)
C0PE12	Hypothetical protein	aQIAtVR	0.57 (Y)	0.98 (N)	0.78 (N)
		aQAEEEtLASER	0.55 (Y)	0.95 (N)	1.06 (N)
K7U162	RNA polymerase ii-associated factor 1 homolog	vEDIDQySEEYsE	0.24 (Y)	0.44 (Y)	0.37 (Y)
		vEDIDQYSEEYsE	0.39 (Y)	1.00 (N)	0.75 (N)
K7V1I2	Arginine serine-rich protein 45-like	gsPSPR	2.72 (Y)	1.33 (N)	2.12 (Y)
		gRsPsPPPk	2.44 (Y)	0.96 (N)	0.98 (N)
Q8W149	Cell division cycle 5-like	eIQTPNPmATPLAsPGPGItPR	(5.15)Y	3.94(Y)	3.58 (Y)
		eIQTPNPmATPLASPGPGItPR	(0.43)Y	0.95 (N)	1.22 (N)
Q9ATN2	NOD26-like membrane integral protein	mQsQLAADEFDTV	0.86 (N)	2.47 (Y)	2.39 (Y)
		rmQsQLAADEFDTV	1.34 (N)	2.51 (Y)	2.69 (Y)

*Y stands for significant changes; N stands for no significant changes. “–”indicates that the phosphorylation level was not detected*.

### Identification of phosphorylation motifs in the phosphopeptides

In the present study, 160, 159, and 161 proteins were identified as having significantly changed phosphorylation levels under drought, heat and their combination, respectively. To determine whether the phosphorylated versions of these proteins shared common phosphorylation site motifs, Motif-X online software (http://motif-x.med.harvard.edu/motif-x.html) was used to predict the motif specificity of these proteins based on the identified phosphorylation sites. An analysis of serine (S), threonine (T) and tyrosine (Y) residues (Table 4) showed that in response to all the three stress treatments, some peptides had the motifs RRx**S** and x**S**Px in common, of which the residues adjacent to the phosphorylated S were enriched for arginine (R) and proline (P); In response to heat stress and the combined stress, the motifs Px**T**P and R**T** were common, of which the residues adjacent to the phosphorylated T were enriched for arginine (P) and proline (R). These results are the first to demonstrate a high sensitivity and specificity for threonine sites under heat and combined drought and heat stress.

**Table 4:** **Phosphorylation motif was analyzed with significant phosphorylation sites in response to D, H, and DH**.

**Stress**	**#**	**Motif**	**Corresponding protein accessions**	**Motif score**	**Foreground matches**	**Foreground size**	**Background matches**	**Background size**	**Fold increase**
D	1	xxxxxxx**R**xx**S**xxxxxxxxxx	C0P9H7; C0PD30; C0PGC2; C0PND4; C4JAN4; K7V0H0; K7TSC5; K7UBY5; K7V5J0; K7VBI0; K7VM50; K7W2Z7; Q41812; B4FI93; B4FRF2; B4FUV7; B6SRZ6; B6T1H0; B6TDN3; B8A0K2; C0HIM6; K7U573; K7V0H0; K7VBA5; K7VBI0; Q9ATN2	12.19	27	94	55507	1013205	5.24
	2	xxxxxxxxxx**SP**xxxxxxxxx	C0HIN5; C0P3W9; C4J1A8; K7U9T7; K7UKJ5; K7UTW7; K7UBY5; K7V1I2; Q8W149; Q9LLI8; B4FAB7; B6SRZ6; B6TDN3; B6TH05; B8A0K2; C4J1A8; K7V0H0; K7UKJ5	9.03	19	67	50073	957698	5.42
	3	xxxxxxxxx**RSA**xxxxxxxxx	C0P3W9; C4IZL5; K7U2X9;	8.90	5	48	2284	907625	41.39
	4	xxxxxx**G**xxx**T**xxxxxxxxxx	C0HF02; C0P3W9; C4J042; C4J4L7; K7TNN3; Q8W149; B6TDN3	4.51	9	27	37040	574595	5.17
H	1	xxxxxxx**R**xx**S**xxxxxxxxxx	BF4F7Z5; BF4F976; B4FI93; B4FRF2; B4FRV0; B4FUV7; B6SRZ6; B6SXN6; B6T1H0; B6T2A6; B6TDN3; B6TQS9; B6TWH2; B6U787; B8A0K2; C0HIM6; C0P3W9; C0P4D8; C0P9L7; C0PCR0; C0PDN0; C0PD30; K7TRK2; K7U573; K7V0H0; K7VBI0; K7VBA5; K7W2Z7; K7WC16; Q41729; Q9ATN2	11.33	31	132	55507	1013205	4.29
	2	xxxxxxxxxx**SP**xxxxxxxxx	B4FAA8; B4FAB7; B4G272; B6SJ48; B6SRZ6; B6SZ69; B6TB14; B6TDN3; B6TH05; B6U8S7; B6UBN4; B7ZYS1; B8A0K2; C0HE50; C0PCR0; C0PHB9; C0PLM8; C4J1A8; C4J9U8; K7UKJ5; K7V0H0; K7V4D9; K7WBH2; Q8W149; Q9LLI8	9.46	25	101	50073	957698	4.54
	3	xxxxxxxx**P**x**TP**xxxxxxxxx	B4F7W7; B6SJ48; C0P3W9; C0PD66	13.09	7	36	2559	574595	43.66
	4	xxxxxxxxx**RT**xxxxxxxxxx	B4FB20; B4FGQ4; B4FQU2; B4FRV0; BF4F7W7; B6SRZ6; C0HE61; C0PLM8; C0PLZ2; K7V4D9; K7VD18	7.28	11	29	27693	572036	7.84
DH	1	xxxxxxx**R**xx**S**xxxxxxxxx	A3KLI0; BF4F976; B4FI93; B4FRF2; B4FUV7; B4FXQ9; B6SJ15; B6SRZ6; B6SXN6; B6T1H0; B6T2A6; B6T346; B6TDN3; B6TWH2; B7ZYP6; B8A0K2; C0HF02; C0HIM6; C0P3W9; C0P4D8; C0P9L7; C0PD30; C0PGC2; K7UBY5; K7U573; K7V0H0; K7VBI0; K7VBA5; Q41729; Q9ATN2	13.40	34	134	55507	1013205	463
	2	xxxxxxxxxx**SP**xxxxxxxxx	BF4F976; B4FAA8; B4FAB7; B6SRZ6; B6TDN3; B6TG30; B6TH05; B6UBN4; B7ZYS1; B8A0K2; C0PHB9; C0PLM8; C4J1A8; K7UKJ5; K7V1I2; K7V0H0; K7V4D9; K7WBH2; Q41735; Q8W149; Q9LLI8	11.13	26	100	50073	957698	4.97
	3	xxxxxxxx**A**x**S**xxxxxxxxxx	B4FAU8; B4FKM0; B4FY62; B4FZ13; B4G272; B6SYC2; B6T649; B6T992; B6TIS5; B7ZYS1; C0P8E4; C0PNT1; F1DJV0; K7TLV1; K7TRK2; K7U5U1; K7UAY1; Q41735; Q5QJA2	3.97	19	74	54021	907625	3.18
	4	xxxxxxxx**P**x**TP**xxxxxxxxx	BF4F7W7; C0P3W9	5.70	7	31	29395	574595	6.31
	5	xxxxxxxxx**RT**xxxxxxxxxx	BF4F7W7; B4FB20; B4FGQ4; B4FQU2; B6SRZ6; C0HE61; C0PLM8; C0PLZ2; K7V4D9	5.44	9	21	26513	545200	7.83

*“x” represents any amino acid*.

### The signaling pathways associated with phosphorylated proteins under various stress conditions

**Figure 5:**
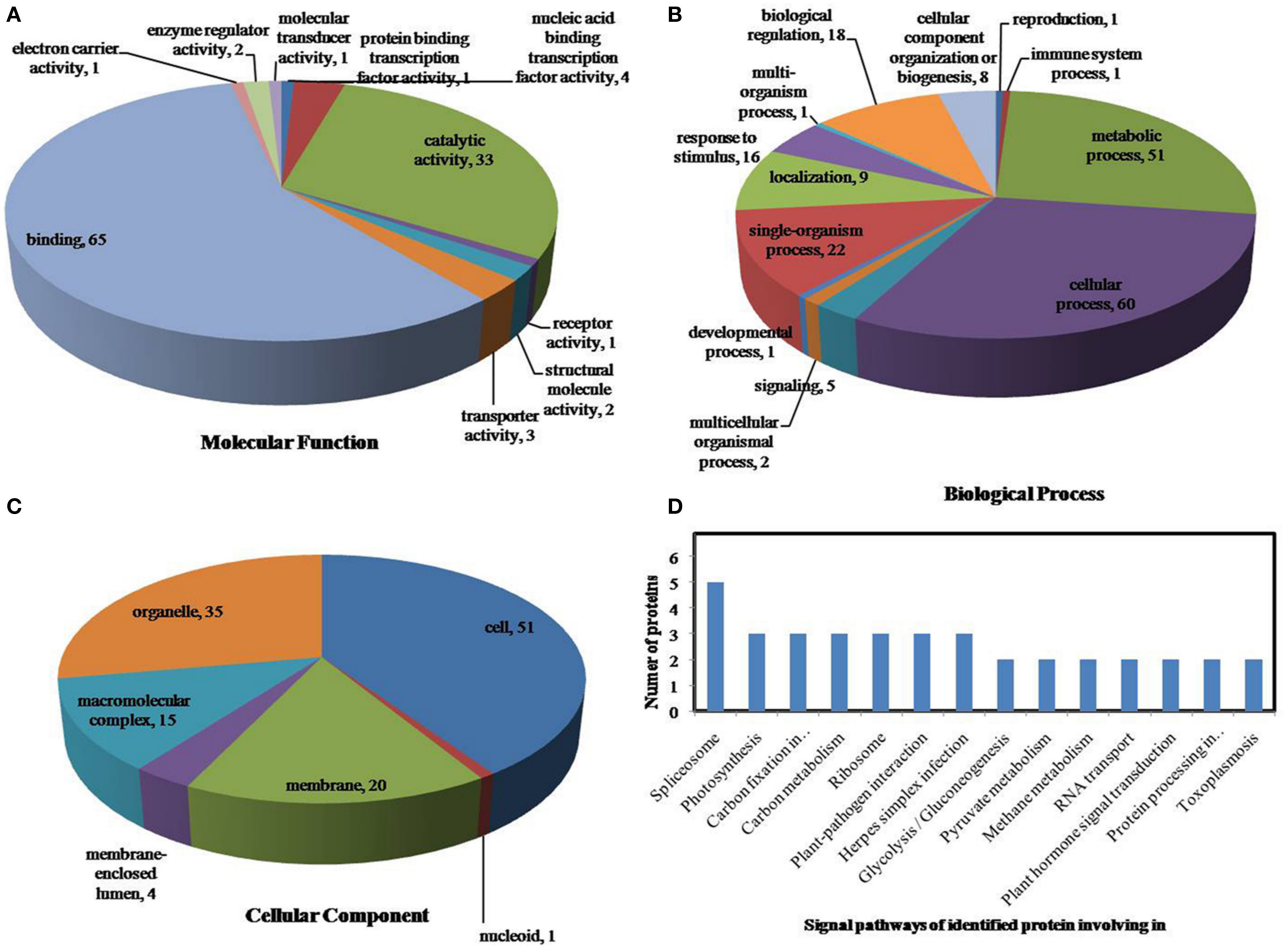
**Pie charts of the distribution of differentially phosphorylated proteins based on their predicted molecular functions (A), biological process (B), and cellular components (C), and the signaling pathways (D) of the proteins identified as involved in drought stress**. There were 160 proteins identified under drought stress in this study, and they were classified according to their known or predicted cellular localization using Blast2Go (http://www.blast2go.com).

**Figure 6:**
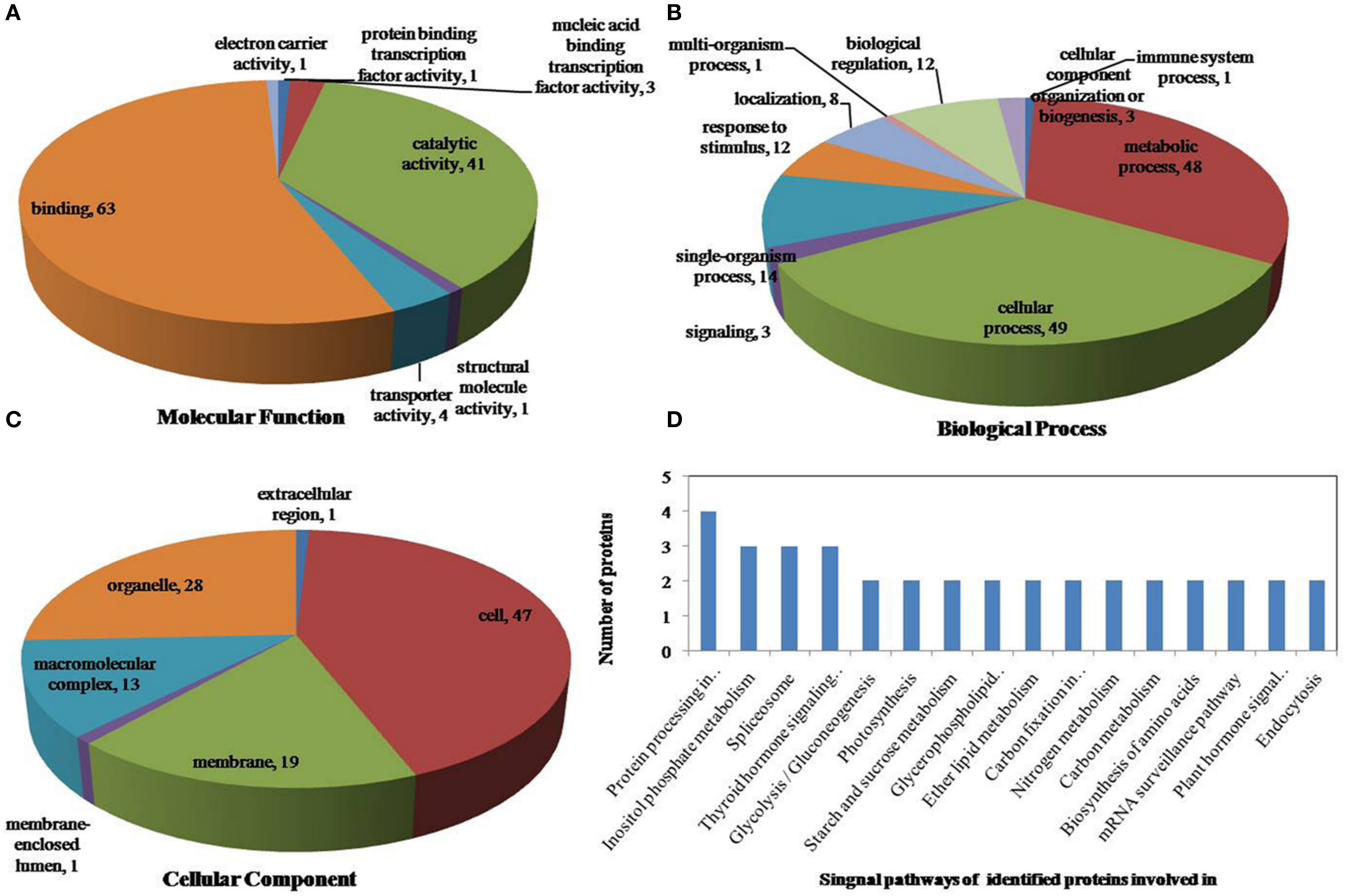
**Pie charts of proteins distribution with differentially phosphorylated peptides according to molecular functions (A), biological process (B), and cellular components (C), and the signaling pathways (D) of identified proteins involved in heat stress**. 159 proteins under drought stress identified in this study were classified according to their known or predicted cellular localization using Blast2Go (http://www.blast2go.com) program, respectively.

**Figure 7:**
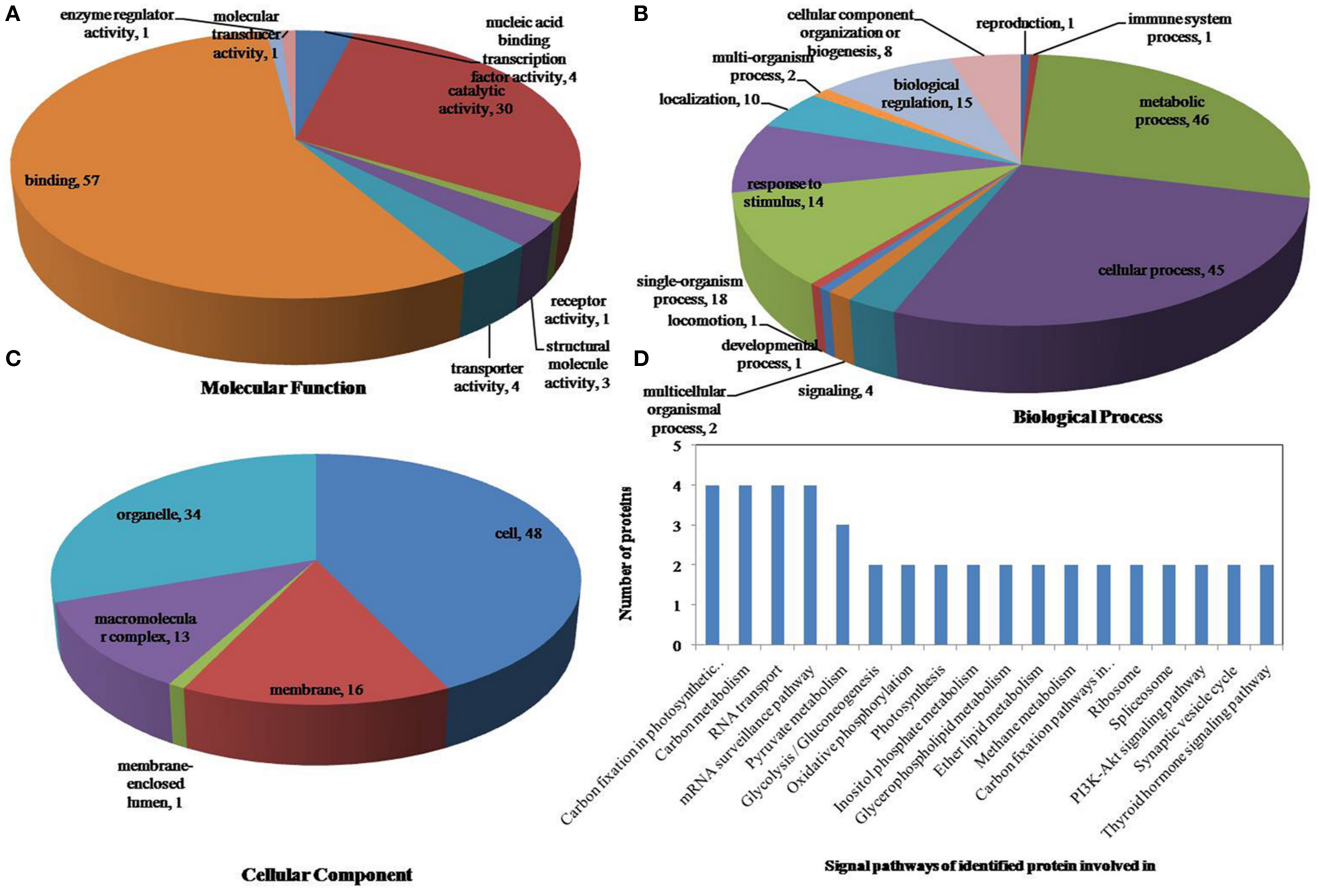
**Pie charts of proteins distribution with differentially phosphorylated peptides according to molecular functions (A), biological process (B), and cellular components (C), and the signaling pathways (D) of identified proteins involved in combined drought and heat stress**. 161 proteins under drought stress identified in this study were classified according to their known or predicted cellular localization using Blast2Go (http://www.blast2go.com) program, respectively.

All identified phosphoproteins were classified using gene ontology (GO) annotation software and were further categorized into three functional groups: molecular function, biological process and cellular component. The results of the GO analyses for drought, heat and the combined stresses are shown in Figures 5–**7**, respectively. The most common molecular functions were binding activity and catalytic activity, and the most common biological processes were cellular process and metabolic process. Moreover, the most common biological processes and molecular functions that the proteins performed were predicted to occur in the organelles, the cytoplasm and the nucleus.

According to biological process analysis using the BLAST2GO program, among these phosphoproteins with significantly changed phosphorylation state, for drought stress, 16 were categorized as “response to stimulus,” 12 were involved in transport and 11 were identified as DNA binding proteins that function as transcription factors (Figure 5B, Table 5); for heat stress, 12 were categorized as “response to stimulus,” nine were transporter proteins, and 14 were DNA binding proteins that function as transcription factors (Figure 6B, Table 5); for the combined stress, nine were categorized as “response to stimulus,” 10 were transporter proteins, and eight were DNA binding proteins that function as transcription factors (Figure 7B, Table 5). In addition, the phosphorylation states of a cell division cycle 5-like, phospholipase C, MYB-CC-type transcription factor, BEL1-type homeodomain protein, bZIP transcription factor superfamily protein, and glycine-rich protein 2 were common for the three stresses; a dnaJ 15-like chaperone protein, disease resistance protein rpp13, and rac GTPase-activating protein 1 were specific to drought; the phosphorylation states of a probable receptor-like protein kinase At5g56460-like, translocase of chloroplast chloroplastic-like isoform x1, uncharacterized LOC100501590, carbohydrate transporter sugar porter transporter, probable metal-nicotianamine transporter ysl12-like, uncharacterized LOC100501590, probable receptor-like protein kinase At5g56460-like, protein stichel-like, hypothetical protein ZEAMMB73_938746 and an expressed methyl binding domain-containing protein were specific to heat stress. 11 proteins were specific to the combined stress (Table 5).

**Table 5:** **Phosphoproteins related to response to stimulus, transport and DNA binding under D, H, and DH respectively**.

**D**	**H**	**DH**
**Protein name/Protein group accessions**	**Protein name/Protein group accessions**	**Protein name/Protein group accessions**
**Response to stimulus**	**Response to stimulus**	**Response to stimulus**
Cell division cycle 5-like/Q8W149	Cell division cycle 5-like/Q8W149	Calcium sensing receptor/B6TVL4
Chaperone protein dnaj 15-like/B4FR07	Heat shock 22 kDa protein/B6T649	Cell division cycle 5-like/Q8W149
Disease resistance protein rpp13/K7UQX6	Heat shock cognate 70 kDa protein 2/B6SZ69	Harpin binding protein 1/Q5QJA2
Heat shock cognate 70 kDa protein 2/B6SZ69	Hypothetical protein ZEAMMB73_026023/K7VD18	Heat shock 22 kDa protein/B6T649
Hypothetical protein/B6SJ48	Hypothetical protein ZEAMMB73_938746/K7V8I9	J domain-containing protein required for chloroplast accumulation response 1-like isoform x2/B6UBN4
Heat shock protein 3/B4G250	J domain-containing protein required for chloroplast accumulation response 1-like isoform x2/B6UBN4	Class I Heat shock protein/BF4F976
Hypothetical protein ZEAMMB73_026023/K7VD18	Class I heat shock protein/BF4F976	Phospholipase c/B4FY17
	Heat shock protein 3/B4G250	Heat shock protein 3/B4G250
Hypothetical protein ZEAMMB73_735596	Phospholipase c/B4FY17	Photosystem ii 10 kda polypeptide/B6SQV5
Phospholipase c/B4FY17	Photosystem II 10 kDa polypeptide/B6SQV5	RAB17 protein/A3KLI0
Protein serine threonine kinase/B6SVK8	Probable receptor-like protein kinase at5g56460-like/C0PHB9	tpa: phototropin family protein kinase/B6SS20
Probable protein phosphatase 2c 31-like/C4J1A8	Translocase of chloroplast chloroplastic-like isoform x1/K7V5E8	
RAB17 protein/A3KLI0	Uncharacterized loc100501590/C4J4G0	
RAC gtpase activating protein 1/B6SWP2		
RPT2-like protein/B6ST41		
tpa: phototropin family protein kinase/B6SS20		
**Transport**	**Transport**	**Transport**
ABC transporter f family member 4-like/K7V5J0	Aquaporin pip2-7/Q9ATM4	Aquaporin pip2-7/Q9ATM4
Ferredoxin- chloroplastic precursor/P27789	Carbohydrate transporter sugar porter transporter/B6U8S7	Leucine-rich repeat-containing protein ddb_g0290504-like
Glycerol 3-phosphate permease/B8A1D5	Hypothetical protein ZEAMMB73_512164/K7UTW7	NOD26-like membrane integral protein/Q9ATN2
Hexose transporter/B6U6U2	Hypothetical protein ZEAMMB73_848936/K7U5U1	Nuclear-pore anchor-like isoform x3/B4FF32
Hypothetical protein ZEAMMB73_512164/K7UTW7	NOD26-like membrane integral protein/Q9ATN2	Photosystem II 10 kda polypeptide/B6SQV5
Hypothetical protein ZEAMMB73_848936/K7U5U1	Probable metal-nicotianamine transporter ysl12-like/E3UJZ2	Plasma membrane H^+^-transporting atpase-like protein/B8A326
Magnesium transporter mrs2-b-like isoform x1/C0PNW3	Probable peptide nitrate transporter at5g13400-like/C0PLZ2	Probable peptide nitrate transporter at5g13400-like/C0PLZ2
Nuclear-pore anchor-like isoform x3/B4FF32	Uncharacterized loc100501590/C4J4G0	tpa: phototropin family protein kinase/B6SS20
Peroxisome biogenesis protein 6-like/K7TNN3		Vacuolar amino acid transporter 1-like/
RAN-binding protein 1/B6T8F4		
**DNA binding**	**DNA binding**	**DNA binding**
At-hook protein 1/B6TI42	At-hook protein 1/B6TI42	At-hook protein 1/B6TI42
bel1-type homeodomain protein/B6SXN6	bel1-type homeodomain protein/B6SXN6	Bel1-type homeodomain protein/B6SXN6
Bzip transcription factor superfamily protein/F1DJV0	Bzip transcription factor superfamily protein/F1DJV0	Bzip transcription factor superfamily protein/F1DJV0
Cell division cycle 5-like/Q8W149	Cell division cycle 5-like/Q8W149	Eukaryotic translation initiation factor isoform 4g-1-like/K7TUM2
SPF1-like DNA-binding protein/B7ZZ27	SPF1-like DNA-binding protein/B7ZZ27	G-box binding factor 1/Q41735
Glycine-rich protein 2/C4JBR4	Glycine-rich protein 2/C4JBR4	Glycine-rich protein 2/C4JBR4
MYB-CC type transfactor/BF4F7W7	Hypothetical protein ZEAMMB73_938746/K7V8I9	MYB-CC type transfactor/BF4F7W7
PHD-finger family homeodomain protein/Q41812	Methyl- binding domain containing expressed/Q94IQ8	Probable receptor-like protein kinase at5g56460-like/C0PHB9
SPF1-like dna-binding protein/B7ZZ27	MYB-CC type transfactor/BF4F7W7	RNA polymerase-associated protein rtf1 homolog/C0P8E4
Transcription initiation factor alpha subunit/C0PBP2	Probable receptor-like protein kinase at5g56460-like/C0PHB9	Transposon protein/B6SRN0
ZF-HD homeobox protein/B4FQM0	Protein stichel-like/K7UDH3	
	SPF1-like dna-binding protein/B7ZZ27	
	Transcription initiation factor alpha subunit/C0PBP2	
	Transposon protein/B6SRN0	

According to KEGG analysis, for drought stress, these phosphoproteins with significantly changed phosphorylation state involved mainly in the protein processing, photosynthesis and carbon metabolism pathways (Figure 5D). There were 11 proteins (group accessions: Q8W149, C0P9H7, B6SL90, C0HIN5, B6SZ69, B4FNM4, B6T1H0, B6UIM2, B4FQU2, K7V1I2, B4G250) involved in protein processing, and seven proteins (group accessions: B4FAW3, B4FRF2, P27789, C0P3W9, E9NQE1, C0PD30, and B4FJG1) involved in photosynthesis and carbon metabolism. For heat stress, these phosphoproteins with significantly changed phosphorylation state involved mainly in signaling pathways related to protein processing, inositol phosphate-glycerophospholipid metabolism and photosynthesis (Figure 6D). Specifically, there were 10 proteins (group accessions: B6SZ69, BF4F976, B6T649, B4G250, BF4F7Z5, Q8W149, B4FQK5, C4J9U8, B6T1H0, and B4FQU2) involved in protein processing, three proteins (group accessions: B6U8P0, B8A1A6, and B4FY17) involved in inositol phosphate-glycerophospholipid metabolism, and five proteins (group accessions: B6SQV5, B4FRF2, C0P3W9, C0PD30, B4FKM0) involved in photosynthesis and carbon metabolism. For drought and heat combined stress, these phosphoproteins with significantly changed phosphorylation state involved mainly in signaling pathways related to photosynthesis and carbon metabolism and protein processing (Figure 7D). In detail, there were eight proteins (group accessions: C0P3W9, B7ZYP6, C0PD30, E9NQE1, B6SKI1, B6SQV5, B7ZYP6, and B4FKM0) involved in photosynthesis and carbon metabolism, and there were eight proteins (group accessions: B6T346, K7TLV1, K7TUM2, K7V1I2, B4FQK5 B6T1H0, B6TG30, K7V792) involved in protein processing. These results indicated that the signaling pathways related to protein processing and to photosynthesis and carbon metabolism played an important role under all three stress conditions. In addition, the differentially phosphorylated proteins that were related to phosphate-glycerophospholipid metabolism were primarily involved in the signaling pathways induced by heat stress.

### Changes in receptor protein phosphorylation

Receptors are proteins that are either embedded in the plasma membrane or localized to the cytoplasm or nucleus of a cell. Receptors enable the body to detect changes in the internal or external environment. In this study, the phosphorylation levels of seven receptor proteins significantly changed under the three stresses. The phosphorylation level of a vacuolar-sorting receptor 3-like protein (B6U4K3) was reduced by the three stress treatments; that of a probable receptor-like protein kinase At5g56460-like (C0PHB9) was reduced by heat and the combined drought and heat stress; that of the TPA: leucine-rich repeat receptor-like protein kinase family protein (B7ZYR5) was reduced by the individual drought and heat stress treatments, that of the receptor-like protein kinase HERK1-like (C0PND4) was increased by all three stress treatments; and that of the calcium-sensing receptor (B6TVL4) and the gibberellin receptor GID1l2 (B6TY90) were increased by the combined stress.

## Discussion

Reversible protein phosphorylation is a ubiquitous regulatory mechanism that plays critical roles in transducing stress signals to bring about coordinated intracellular responses. In this study, we report the comprehensive analysis of the phosphorylation changes in maize leaves response to drought, heat and their combination using iTRAQ-based quantitative proteomic and LC-MS/MS methods.

### Phosphorylation regulatory network in maize leaves response to three stress treatments

The interplay between phosphatases and kinases strictly controls biological processes such as metabolism, transcription, cell cycle progression, differentiation, cytoskeletal arrangement and cell movement, apoptosis, intercellular communication, and immunological functions (Johnson, 2009; Pjechová et al., 2014). In this study, five kinases and three phosphatases were identified under heat stress, three kinases and two phosphatases were under drought stress, and three kinases and three phosphatases were under combined heat and drought stress. Nevertheless, according to the analysis of protein-protein interactions among significantly changed phosphoproteins, only AGC_PKAPKG_like.1-ACG kinases, phospholipase C and protein phosphatase 2C were found to have interactions with some phosphoproteins, which involved in gene expression, protein synthesis, and stress tolerance. Our analysis suggested potential multiple phosphorylation regulatory mechanisms of these phosphoproteins for further experimental validation. Furthermore, phospholipase C and protein phosphatase 2C were showed to have a significant phosphoryltion changes under three stress conditions. Previous study also show that the phosphorylation levels of phospholipase C and protein phosphatase 2C is obviously affected (Li et al., 2013; Hwang et al., 2014; Wei et al., 2014; Zhang et al., 2014), which indicated that the signal pathway related to phospholipase C and protein phosphatase 2C played an important role in plants response to stress.

Besides, we found phosphorylation events in other important kinase. For example, serine threonine-protein kinase wnk4-like (K7TZQ1) changed significantly under three stress conditions. Ser/Thr phosphorylation plays key roles in the regulation of plant growth and development. In wheat response to drought stress, phosphoproteome analysis also reveals two serine threonine-protein kinases (Zhang et al., 2014). Calcium-dependent protein kinase (C4J038, CDPK) changed under drought stress. In plants, calcium is a ubiquitous second messenger in signal transduction cascades. Most of the known Arabidopsis calcium-stimulated protein kinase activities are related to CDPKs (Cheng et al., 2002). In wheat, among nine phosphorylated CDPKs identified, CDPK7 was phosphorylated in response to drought stress (Lv et al., 2014).

### Enzymes involved in the gluconeogenesis pathway

Phosphoenolpyruvate carboxykinase (PEPCK, C0P3W9) plays an important role in organic acid metabolism. In the cytoplasm, PEPCK and malate dehydrogenase can synthesize malate from glycolytically derived phosphoenolpyruvate (Fortes et al., 2011). In this study, PEPCK had 7 significantly phosphorylated peptides under heat stress, 5 under drought stress and 8 under the combined stress. Regardless of drought, heat and their combination, the phosphorylation level of these peptides increased or decreased under the same stress. Importantly, although the phosphorylation level changed across the different treatments, the protein expression level did not. Similarly, in drought-stressed *Pinus halepensis*, PEPCK activity increased without an increase in its transcription or translation (Fontaine et al., 2003; Hýsková et al., 2014). These results demonstrated that PEPC phosphorylation plays an important role in plants' responses to abiotic stress.

Fructose-bisphosphate aldolase is a key enzyme in the pathways of gluconeogenesis. In drought-tolerant tomato response to drought, the gene encoding this enzyme is down-regulated by drought stress (Gong et al., 2010). In the present study, the three stresses all increased the phosphorylation level of this enzyme, and heat and combined drought and heat stress decreased the protein expression. Gluconeogenesis consumes plenty of energy thus down-regulation of the fructose-bisphosphate aldolase could inhibit gluconeogenesis for keeping energy in stressed plants.

### Protein degradation by ubiquitination

Protein degradation via the ubiquitin/26S proteasome system is the main protein degradation pathway; it plays a crucial role in removing misfolded or damaged proteins and in controlling the abundance of certain regulatory proteins during abiotic stress (Vierstra, 2003; Stone, 2014). Ubiquitination is a multi-step process involving the sequential action of three enzymes: E1 (ubiquitin activating enzyme), E2 (ubiquitin conjugating enzyme), and E3 (ubiquitin ligase). It has been shown that in plants, certain E3 ubiquitin ligases are involved in transcription-dependent resistance to high temperature and drought stress (Kim and Kim, 2013; Liu et al., 2014). *Saccharomyces* bearing a mutation in RSP5, which encodes an essential E3 ubiquitin ligase, are hypersensitive to heat stress (Uesugi et al., 2014). In humans, DNA damage induces the phosphorylation-dependent degradation of the E3 ubiquitin ligase TRIM24 in the nucleus, which disrupts the interaction between TRIM24 and p53 and activates p53 (Jain et al., 2014). However, in the present study, the E3 ubiquitin ligases rglg2-like isoform x1 and upl4-like were dephosphorylated under heat stress and the combined drought and heat stress, but the expression level of rglg2-like changed only minimally (upl4-like was not identified in the protein expression analysis). These results indicate that decreasing the phosphorylation level of the 26S proteasome protein complex can promote the eventual degradation of regulatory proteins that are involved in heat stress.

#### Phosphoproteins involved in water, sugar and H^+^ transport

Transporter proteins are important for turgor pressure maintenance and water potential regulation, which are crucial for the growth and survival of plants under biotic and abiotic stress. For example, plasma membrane intrinsic proteins (PIPs) are primary channels that mediate water uptake in plant cells, and they are regulated by phosphorylation. In this study, two aquaporin isoforms were differentially phosphorylated under heat and the combined stress: phosphorylation of the aquaporin PIP2-7 and the integral membrane protein nod26-like were reduced and increased, respectively. Other results also demonstrated that the aquaporin CsPIP2-1, which is involved in phosphorylation-dependent salt- and drought-stress responses in Camelina (Jang et al., 2014), and PIPs from maize shoots are phosphorylated on serine residues by a calcium-dependent kinase both *in vitro* and *in vivo* (Van Wilder et al., 2008). Together, these results indicate that phosphorylation plays an important role in the activity of PIPs and, consequently, of water channels.

H^+^-ATPase, which belongs to the cation transport ATPase (P-type) family, is related to H^+^ electrochemical gradient across the plasma membrane, which regulates cell growth and response to stress (Schaller and Oecking, 1999; Bobik et al., 2010). In *Nicotiana tabacum*, the phosphorylation of the H^+^-ATPases leads to the increase of this enzyme activity (Bobik et al., 2010). In wheat, the H^+^-ATPase identified in two cultivars showed up-regulated phosphorylation levels (Zhang et al., 2014). In our study, the significant phosphorylation (B8A326) was found only under the drought and heat combined stress. Taken together, these results show that H^+^-ATPase may enhance the activity itself and participate in H^+^ homeostasis related to osmotic regulation. Besides, glycerol 3-phosphate permease (B8A1D5) and hexose transporter (B6U6U2) showed down-regulated and up-regulated phosphorylation level, respectively under drought stress. Carbohydrate transporter sugar porter transporter (B6U8S7) showed up-regulated phosphorylation level under heat stress. Taken together, these results showed that the phosphorylation and dephosphorylation of transporters might help cell to maintain cell solute and ion stability which might play an active role in plant adaptation to abiotic stress.

### The sites of phosphorylation differ across the three stress treatments

Analysis of phosphorylation site patterns showed a striking distinction among the three stress treatments, indicating that drought, heat, and the combined drought and heat stress resulted in different sites being phosphorylated (Table 1, Tables S1–S6). Under the three stress treatments, the phosphopeptides of each protein had different phosphorylation levels and regulatory patterns under the three stress treatments (Table 3).

Further, analysis of these motifs for significant phosphorylation phosphoproteins showed that [SP] and [RxxS] shared in common under three stress treatments, which were showed. [GxxxT] and [RSA] were specific to drought stress. [PxTP] and [RT] shared in common under heat stress and combined double stresses. [AxS] were specific to combined double stresses. These results provide substantial novel insight into phosphorylation and dephosphorylation and signal transduction; this is the first study to dissect the differences between drought, heat and combined drought and heat stress. Importantly, these results reveal a previously unappreciated quantity and diversity of phosphorylated protein isoforms, and uncover novel signaling pathways.

### Phosphorylation changes of heat shock proteins

Heat shock proteins (HSPs) have “chaperone-like” activities and accumulate in response to various stresses (Eisenhardt, 2013). At least five families of chaperones are found in higher plants: the HSP100 (Clp) family, the HSP90 family, the HSP70 (DnaK) family, chaperonins (GroEL and HSP60), and the small (20–40 kD) HSP (sHSP) family. sHSPs play important and extensive roles in plant defenses against abiotic stress (Mu et al., 2013). The phosphorylation of sHSPs has been demonstrated in maize mitochondria (Lund et al., 2001) and in barley (*Hordeum vulgare*) seed endosperm (Slocombe et al., 2004). In this study, the phosphorylation level of seven HSPs, including five sHSPs (B4G250, B4F976, B6T649, C0P2N6, and B4FR07) and two HSP70s, changed significantly under drought, heat or combined drought and heat stress. Notably, under heat stress and combined drought and heat stress, the phosphorylation level of the identified sHSPs (except B4FR07) was up-regulated. Taken together, these results indicate that the phosphorylation of sHSPs appears to be important for the regulation of sHSP function in plant responses to heat and to combined drought and heat stress. These results are the first to demonstrate the phosphorylation of plant sHSPs under the combination of drought and heat stress.

### Receptor proteins

Most proteins that are synthesized on the rough endoplasmic reticulum are delivered to various cellular destinations, including the vacuoles and lysosomes. Such sorting involves the recognition of targeting signals on the proteins by receptors. The vacuolar-sorting receptor (VSR) is involved in sorting clathrin-coated vesicles from the Golgi apparatus to the vacuoles. In pumpkin, a putative vacuolar sorting receptor, PV72, undergoes a Ca^2+^-dependent conformational change (Watanabe et al., 2002). Here, the phosphorylation level of vacuolar sorting receptor 3-like (B6U4K3) was significantly reduced under the three stress treatments, suggesting that stress affects vacuolar sorting in higher plants. The calcium-sensing receptor modulates the cytoplasmic Ca^2+^ concentration and is crucial for proper stomatal regulation in response to elevated levels of external Ca^2+^. In this study, a calcium-sensing receptor (B6TVL4) located in the chloroplast thylakoid membrane had significantly increased phosphorylation levels under the combined drought and heat stress, but little is known about the role of calcium-sensing receptors in plant responses to stress. Gibberellin (GA) perception is mediated by GID1 (GA-INSENSITIVE DWARF1), a receptor that shows similarity to hormone-sensitive lipases. The discovery of GID1 has yielded new insight into how GA is perceived (Hirano et al., 2008). In this study, the phosphorylation level of the gibberellin receptor GID1L2 (B6TY90) clearly increased under combined drought and heat stress. However, at present, the molecular mechanism of GID1L2's function in GA signaling, and especially in GA-dependent regulation of plant responses to stress, is unclear. The probable receptor-like, At5g56460-like protein kinase; a TPA: leucine-rich repeat receptor-like protein kinase family protein; and the receptor-like protein kinase HERK1-like were also identified, although their mechanisms of action require further study.

## Conclusions

Compared to single drought or heat stress, their combination had a different effect on phosphorylation sites and level of phosphoproteins. Of the 282 phosphoproteins identified in the present study, only 46 were found to show similar changes under all stress conditions. Of the 12 phosphorylation motifs identified, only two were common under the three stresses. In particular, these phosphoproteins involved in signaling pathways were different among drought, heat and their combination. Therefore, our results could not only provide new information for understanding the mechanisms of crop tolerance to the combined stress, but also contribute to the identification of crop cultivars with increased tolerance to increasing climate variability.

## Author contributions

XH and WW conceived and designed the research. FZ and NL performed the experiments, DZ, GZ and XH analyzed the data. LW and CL contributed reagents/materials/analysis tools. XH and DZ wrote the paper. All authors read and approved the manuscript.

### Conflict of interest statement

The authors declare that the research was conducted in the absence of any commercial or financial relationships that could be construed as a potential conflict of interest.
