# Supplementary material for: Phosphoproteomic analysis of the response of maize leaves to drought, heat and their combination stress
Source: Front Plant Sci. 2015 May 5;6:298. doi: 10.3389/fpls.2015.00298 (PMC4419667; doi:10.3389/fpls.2015.00298)
Supplement: Supplementary file 1 [file Table1.DOC]

**Table S1 ︱The proteins with significant phosphorylation level changes under H and DH.**

| **Protein Group Accessions** | **Protein name** | **Sequence** | **PhosphoRS Site Probabilities (listed >75%)** | **Ratio of phosphorylation level** | | | **P-Value** | | |
| --- | --- | --- | --- | --- | --- | --- | --- | --- | --- |
|  |  |  |  | **D/CK** | **H/CK** | **DH/CK** | **D/CK** | **H/CK** | **DH/CK** |
| B4F8M5 | **Octicosapeptide phox bem1p domain-containing protein** | sADQsVETGk | S(1): 100.0; S(5): 100.0 | 1.40 | 1.85 | 1.82 | 0.2781 | 0.0284 | 0.0773 |
| B4F976 | **17.4 kDa class I heat shock protein 3** | nVQItG | T(5): 100.0 | 0.79 | 5.76 | 9.40 | 0.2580 | 0.0000 | 0.0000 |
| B4G250 | **Heat shock protein** | tSsETAAFAGAR | S(3): 98.4 | 0.90 | 4.39 | 8.52 | 0.5853 | 0.0000 | 0.0000 |
| B4FAA8 | **Transmembrane protein 115-like** | lAADNAGDTEAsPR | S(12): 100.0 | 1.02 | 0.39 | 0.53 | 0.9743 | 0.0010 | 0.0401 |
| B4FAU8 | **Uncharacterized protein LOC100191974** | sAFGDGNR | S(1): 100.0 | 0.84 | 0.55 | 0.43 | 0.3737 | 0.0351 | 0.0069 |
| B4FB20 | **Wat1-related protein at5g64700-like** | tAAGVAk | T(1): 100.0 | 0.74 | 0.54 | 0.47 | 0.1348 | 0.0318 | 0.0162 |
| B4FF32 | **Nuclear-pore anchor-like isoform x3** | aAVEPDQsPITQPGAADASPsR | S(8): 100.0 | 1.02 | 1.65 | 2.38 | 0.0245 | 0.1395 | 0.3068 |
| B4FQ49 | **Clavaminate synthase-like protein at3g21360-like** | vLGEGDDPsSPIGR | S(9): 78.0 | 0.93 | 3.24 | 5.64 | 0.6982 | 0.0000 | 0.0000 |
| B4FUV7 | **Uncharacterized protein LOC100275458** | sGsVTNWTSANR | S(3): 100.0 | 0.82 | 0.33 | 0.34 | 0.3202 | 0.0001 | 0.0005 |
| B4FUV7 | **Uncharacterized protein LOC100275458** | sVSSWSTPPAPPPVQR | S(1): 98.7 | 0.94 | 0.62 | 0.54 | 0.7366 | 0.1020 | 0.0483 |
| B4FXH5 | **SR-related ctd associated factor 6** | hVSPDPtTFSGSAPVPSk | T(7): 80.0 | 1.43 | 2.05 | 1.62 | 0.2507 | 0.0107 | 0.1561 |
| B4FY62 | **TPA: c3hc zinc finger-like family protein** | aDsVEsGEk | S(3): 100.0; S(6): 100.0 | 0.74 | 0.44 | 0.45 | 0.1471 | 0.0040 | 0.0099 |
| B4G272 | **Non-green plastid inner envelope membrane protein** | iAAGsPk | S(5): 100.0 | 0.74 | 0.57 | 0.54 | 0.1365 | 0.0493 | 0.0497 |
| B6SJ15 | **Protein fam32a-like** | sEsPIDPNNER | S(3): 100.0 | 1.12 | 1.67 | 2.12 | 0.7247 | 0.0666 | 0.0273 |
| B6SKI1 | **Photosystem I reaction center subunit II** | atAGEAVTEEAPk | T(2): 100.0 | 1.35 | 1.69 | 2.01 | 0.3386 | 0.0610 | 0.0404 |
| B6SQV5 | **Photosystem II 10 kDa polypeptide** | gVyQFVDk | Y(3): 100.0 | 1.17 | 1.82 | 2.31 | 0.6271 | 0.0321 | 0.0138 |
| B6SRN0 | **Transposon protein** | lALPSTGGtDNDGEGTIER | T(9): 98.2 | 0.89 | 0.32 | 0.34 | 0.5597 | 0.0000 | 0.0005 |
| B6SRZ6 | **Ubiquitin-protein ligase** | eLGVsscGNTAEF | S(6): 97.2 | 1.03 | 0.65 | 0.53 | 0.9313 | 0.1322 | 0.0384 |
| B6SU00 | **Lim domain-containing protein** | aAEAQPEQPASDssS | S(13): 94.4 | 0.73 | 0.47 | 0.26 | 0.8362 | 0.5375 | 0.9897 |
| B6SWV8 | **Crs2-associated factor 1** | vGDDAsGDER | S(6): 100.0 | 0.88 | 0.52 | 0.32 | 0.5167 | 0.0229 | 0.0003 |
| B6SXN6 | **Bel1-type homeodomain protein** | gAsASNPNNNPGNk | S(3): 100.0 | 1.11 | 0.37 | 0.37 | 0.7422 | 0.0004 | 0.0013 |
| B6SYC2 | **Protein plastid movement impaired 2-like** | eAEAAAEAEsAAGEQk | S(10): 100.0 | 0.77 | 0.64 | 0.54 | 0.2011 | 0.1225 | 0.0444 |
| B6T1H0 | **TPA: 40s ribosomal protein s9** | rSEsLAk | S(4): 100.0 | 1.33 | 0.53 | 0.49 | 0.3650 | 0.0269 | 0.0219 |
| B6T2A6 | **Stem-specific protein tsjt1-like** | vDsEGAmcGATFk | S(3): 100.0 | 0.99 | 0.36 | 0.52 | 0.9270 | 0.0003 | 0.0351 |
| B6T346 | **Tho complex subunit 4** | gGsAPGGGSGGVGPTR | S(3): 100.0 | 0.82 | 0.60 | 0.47 | 0.3230 | 0.0808 | 0.0136 |
| B6T649 | **Heat shock 22 kDa protein** | yEGAEsEDDSVR | S(7): 100.0 | 0.86 | 5.84 | 7.82 | 0.7918 | 0.9060 | 0.5063 |
| B6T8V1 | **RNA-binding protein fus-like** | nDtPISQNEIR | T(3): 100.0 | 1.67 | 2.64 | 2.30 | 0.0976 | 0.0006 | 0.0141 |
| B6TB14 | **Splicing arginine serine-rich 6** | sGSPtGSksP | S(3): 100.0; S(9): 99.9 | 1.39 | 2.50 | 3.03 | 0.2886 | 0.0012 | 0.0011 |
| B6TDN3 | **Uncharacterized protein LOC100276535** | aGtPPIsPAEALDR | T(3): 100.0; S(7): 100.0 | 1.49 | 2.17 | 2.50 | 0.2005 | 0.0060 | 0.0071 |
| B6THU8 | **Sun domain-containing protein 2-like** | sNYsSEGR | S(4): 83.3 | 0.77 | 0.55 | 0.42 | 0.1967 | 0.0351 | 0.0057 |
| B6TIS5 | **Somatic embryogenesis related protein** | dAAQsADPPNAESk | S(5): 100.0 | 0.94 | 0.44 | 0.36 | 0.7637 | 0.0036 | 0.0009 |
| B6TNE3 | **Uncharacterized protein LOC100277061** | sAsSPLLNcGR | S(3): 100.0 | 0.67 | 0.47 | 0.39 | 0.0516 | 0.0082 | 0.0026 |
| B6TQS9 | **Uncharacterized protein LOC100277204** | sQscVGDAAGR | S(3): 100.0 | 0.86 | 1.78 | 1.88 | 0.4478 | 0.0394 | 0.0628 |
| B6TWH2 | **Soluble inorganic pyrophosphatase** | iLsSLSR | S(3): 100.0 | 1.31 | 1.92 | 2.33 | 0.3903 | 0.0200 | 0.0126 |
| B6U8P0 | **Loc100285723 precursor** | wFASVPTsTQPNR | S(4): 99.6 | 0.72 | 0.40 | 0.35 | 0.1124 | 0.0014 | 0.0008 |
| B6UBN4 | **J domain-containing protein required for chloroplast accumulation response 1-like isoform x2** | lFSPEssPk | S(6): 99.9; S(7): 97.4 | 1.11 | 0.51 | 0.44 | 0.7533 | 0.0184 | 0.0075 |
| B7ZYP6 | **Pyruvate orthophosphate dikinase** | sDsGAGR | S(3): 100.0 | 0.92 | 0.64 | 0.54 | 0.6714 | 0.1212 | 0.0497 |
| B7ZYS1 | **TPA: phototropic-responsive nph4 family protein** | eAPTSSFYGGEsPTPAPSLQGR | S(12): 97.5 | 0.68 | 0.41 | 0.49 | 0.0608 | 0.0019 | 0.0227 |
| B7ZYS1 | **TPA: phototropic-responsive nph4 family protein** | eAPTSSFYGGEsPTPAPsLQGR | S(18): 91.7 | 1.00 | 0.28 | 0.53 | 0.9656 | 0.0000 | 0.0384 |
| B8A1A6 | **Phosphoesterase family protein** | wFASVPSStQPNR | T(9): 97.3 | 0.81 | 0.54 | 0.47 | 0.3005 | 0.0345 | 0.0153 |
| C0HH76 | **Unknown** | lSSsSPSAAAASR | S(3):81.8 | 1.39 | 2.69 | 2.52 | 0.2875 | 0.0005 | 0.0065 |
| C0P2N6 | **Chaperone protein dnaj 10 isoform 1** | vETSTDQQAASsH | S(12): 97.4 | 0.90 | 0.26 | 0.21 | 0.6112 | 0.0000 | 0.0000 |
| C0P3W9 | **Phosphoenolpyruvate carboxykinase** | sAPttPIk | S(1): 98.5 | 0.70 | 0.35 | 0.22 | 0.0850 | 0.0002 | 0.0000 |
| C0P3W9 | **Phosphoenolpyruvate carboxykinase** | sAPTTPIk | S(1): 100.0 | 1.12 | 0.49 | 0.49 | 0.7334 | 0.0119 | 0.0201 |
| C0P3W9 | **Phosphoenolpyruvate carboxykinase** | gEAAAQGAPStPR | T(11): 98.4 | 0.90 | 0.47 | 0.51 | 0.6037 | 0.0082 | 0.0320 |
| C0P3W9 | **Phosphoenolpyruvate carboxykinase** | eVDYADNsVTENTR | S(8): 91.1 |  | 4.83 | 3.43 | 1.0000 | 0.0000 | 0.0003 |
| C0P4D8 | **Dynamin-2b-like isoform 2** | qLsIHDNR | S(3): 100.0 | 1.14 | 1.75 | 1.98 | 0.6758 | 0.0462 | 0.0442 |
| C0P4D8 | **Dynamin-2b-like isoform 2** | aSsPQTDAEQGGGSLk | S(3): 80.0 | 0.79 | 2.02 | 1.85 | 0.2554 | 0.0123 | 0.0693 |
| C0P9L7 | **E3 ubiquitin-protein ligase rglg2-like isoform x1** | ssSFSQQSGVYSR | S(2): 79.0 | 0.88 | 0.42 | 0.40 | 0.5096 | 0.0022 | 0.0033 |
| C0PE44 | **Branchpoint-bridging protein** | wEQSNDDSANNsGGEGGAVGR | S(12): 100.0 | 0.93 | 0.48 | 0.49 | 0.6943 | 0.0114 | 0.0215 |
| C0PHB5 | **tRNA (guaninen1)-methyltransferase-like** | gGLDDNDGAAEGAADGDHsEEDLNk | S(19): 100.0 | 1.13 | 1.78 | 2.30 | 0.7074 | 0.0396 | 0.0140 |
| C0PHB9 | **Probable receptor-like protein kinase at5g56460-like** | vQsPSEVDR | S(3): 100.0; | 0.91 | 0.44 | 0.45 | 0.6186 | 0.0042 | 0.0103 |
| C0PLM8 | **Unknown** | tTLNAGENsPTTNGVTVLGAR | S(9): 93.8 | 0.75 | 0.53 | 0.49 | 0.1526 | 0.0288 | 0.0212 |
| C0PLQ0 | **Hypothetical protein** | sSsVISVAPsASELAAGmGREDGR | S(1): 83.3 | 1.18 | 2.66 | 2.23 | 0.6056 | 0.0005 | 0.0183 |
| C0PLZ2 | **Probable peptide nitrate transporter at5g13400-like** | aRtPLGAAYEPPSAAAGGGGTtPVNIR | T(3): 100.0 | 0.85 | 0.56 | 0.42 | 0.4149 | 0.0444 | 0.0046 |
| C0PNT1 | **Glycine-rich rna-binding protein 2** | nITVNEAQsR | S(9): 100.0 | 1.37 | 1.71 | 2.08 | 0.3150 | 0.0548 | 0.0309 |
| C4J9J4 | **KH domain-containing expressed** | lASDEGLGNEDsEEISPQVTVR | S(3): 97.6 | 0.85 | 0.53 | 0.54 | 0.4345 | 0.0259 | 0.0464 |
| E3UJZ2 | **Probable metal-nicotianamine transporter ysl12-like** | nSTLPVsNNGSPITEGVSFDDER | S(7): 98.1 | 0.83 | 2.12 | 1.73 | 0.3554 | 0.0075 | 0.1084 |
| K7TRK2 | **Hypothetical protein ZEAMMB73_626728** | aNsQVsNLkLAGTtADVsk | S(3): 75.2; S(6): 75.2; T(13): 75.2; T(14): 75.2; S(18): 99.3 | 1.00 | 1.88 | 1.53 | 0.9924 | 0.0242 | 0.2150 |
| K7U5V4 | **TPA:RNA-binding zinc finger family protein** | lGPAAFPYSPTGSLPGSPsAASk | S(19): 83.7 | 0.77 | 0.32 | 0.32 | 0.2077 | 0.0001 | 0.0002 |
| K7UAY1 | **Serine threonine-protein kinase ctr1** | dIASsTER | S(5): 79.6 | 1.13 | 1.65 | 2.30 | 0.7031 | 0.0714 | 0.0143 |
| K7V4D9 | **E3 ubiquitin-protein ligase upl4-like** | tAVNPEANDsPR | S(10): 100.0 | 0.95 | 0.44 | 0.43 | 0.7793 | 0.0037 | 0.0066 |
| K7VH05 | **Hypothetical protein ZEAMMB73_826358** | stGGAGRGSAQGR | T(2): 99.8 | 0.70 | 0.48 | 0.27 | 0.0802 | 0.0096 | 0.0000 |
| K7WBH2 | **Bag family molecular chaperone regulator 6-like** | iSEVSPQsPk | S(8): 100.0 | 0.95 | 2.89 | 3.15 | 0.7715 | 0.0002 | 0.0007 |
| K7WCY0 | **Hypothetical protein ZEAMMB73_644677** | fQQQPsGR | S(6): 100.0 | 1.47 | 1.96 | 2.15 | 0.2156 | 0.0169 | 0.0245 |
| Q41729 | **Carbonic anhydrase** | lTsGFQQFk | T(2): 98.8 | 1.12 | 2.38 | 2.37 | 0.7356 | 0.0022 | 0.0111 |
| Q9ATM4 | **Aquaporin pip2-7** | aLGsFRsNA | S(7): 100.0 | 1.03 | 0.40 | 0.46 | 0.9439 | 0.0014 | 0.0129 |
| Q9ATN2 | **NOD26-like membrane integral protein** | mQsQLAADEFDTV | S(3): 100.0 | 0.86 | 2.47 | 2.39 | 0.4681 | 0.0014 | 0.0102 |
|  | **NOD26-like membrane integral protein** | rmQsQLAADEFDTV | S(4): 100.0 | 1.34 | 2.51 | 2.69 | 0.3485 | 0.0011 | 0.0036 |

**Note: CK**: control; **D**: drought stress; **H**: heat stress; **DH**: combined drought and heat stress.
