# Supplementary material for: Phosphoproteomic analysis of the response of maize leaves to drought, heat and their combination stress
Source: Front Plant Sci. 2015 May 5;6:298. doi: 10.3389/fpls.2015.00298 (PMC4419667; doi:10.3389/fpls.2015.00298)
Supplement: Supplementary file 2 [file Table2.DOC]

**Table S2︱The Proteins with significant phosphorylation level changes only under D and DH.**

| **Protein Group Accessions** | **Protein name** | **Sequence** | **PhosphoRS Site Probabilities (>75%)** | **Ratio of phosphorylation level** | | | **P-Value** | | |
| --- | --- | --- | --- | --- | --- | --- | --- | --- | --- |
|  |  |  |  | D/CK | H/CK | DH/CK | D/CK | H/CK | DH/CK |
| A3KLI0 | **RAB17 protein** | sGsSSSSSSEDDGmGGR | S(3): 100.0 | 9.44 |  | 10.54 | 0.0000 | 1.0000 | 0.0000 |
| B4FFI8 | **Membrane-anchored ubiquitin-fold protein 3-like** | nsGIADNFSLLFISFsWLP | S(2): 80.0; S(16): 80.0 | 2.58 | 0.82 | 1.72 | 0.0022 | 0.5115 | 0.1099 |
| B4G0P6 | **Uncharacterized membrane protein at1g16860-like** | nTsFGGAASNsGPVSNAVGR | S(3): 96.3; S(11): 100.0 | 0.62 | 0.81 | 0.59 | 0.0188 | 0.4754 | 0.0919 |
| B6SS20 | **TPA: phototropin family protein kinase** | gRDsGVGSTR | S(4): 100.0 | 0.46 | 0.76 | 0.53 | 0.0002 | 0.3555 | 0.0413 |
| B6TI42 | **At-hook protein 1** | aPNTAPSAsPDGAk | S(9): 100.0 | 1.89 | 1.32 | 1.86 | 0.0409 | 0.3097 | 0.0674 |
| B6T890 | **Nucleolin-like isoform x1** | eNsNLIDADDSDELR | S(3): 50.0; S(11): 50.0 | 0.20 | 0.83 | 0.29 | 0.0000 | 0.5313 | 0.0001 |
| B6TZS3 | **Uncharacterized protein LOC100277773** | gEGmGSAVADSGEsR | S(14): 99.9 | 2.51 | 1.40 | 2.69 | 0.0029 | 0.2232 | 0.0036 |
| B6UIM2 | **40s ribosomal protein s10** | gSAPADFQPsFR | S(10): 100.0 | 1.98 | 1.54 | 1.89 | 0.0274 | 0.1184 | 0.0619 |
| C0HF02 | **Chlorophyll a-b binding protein chloroplastic-like** | fEsSEVk | S(3): 98.3 | 1.55 | 1.12 | 2.41 | 0.1615 | 0.6416 | 0.0096 |
| C0P3W9 | **Phosphoenolpyruvate carboxykinase** | rsAPTtPIk | S(2): 100.0; T(6): 100.0 | 0.66 | 0.67 | 0.51 | 0.0406 | 0.1725 | 0.0286 |
| C0P3W9 | **Phosphoenolpyruvate carboxykinase** | sAPStPkR | T(5): 97.7 | 2.04 | 1.38 | 1.71 | 0.0216 | 0.2387 | 0.1142 |
| C0P8E4 | **RNA polymerase-associated protein rtf1 homolog** | aGsPPSDGSNDGNNR | S(3): 100.0 | 1.50 | 0.93 | 2.42 | 0.1937 | 0.8551 | 0.0094 |
| C0PEW7 | **Vacuolar amino acid transporter 1-like** | sLIIQsDDDDDAR | S(6): 100.0 | 1.65 | 1.28 | 2.22 | 0.1066 | 0.3606 | 0.0187 |
| C0PGC2 | **TPA: Zn-containing protein** | rPGsPPPQR | S(4): 100.0 | 0.64 | 0.80 | 0.52 | 0.0297 | 0.4591 | 0.0373 |
| C4IZL5 | **DNA binding protein** | sAGsADEDDDGPASGSGR | S(4): 99.7 | 1.91 | 0.58 | 1.52 | 0.0363 | 0.0620 | 0.2158 |
| C4J1A8 | **Probable protein phosphatase 2c 31-like** | eQSSPTSNLsPR | S(4): 99.7; S(10): 99.8 | 3.43 | 1.00 | 2.33 | 0.0001 | 0.9608 | 0.0127 |
| E9NQE1 | **Phosphoenolpyruvate carboxylase** | hHsIDAQLR | S(3): 100.0 | 2.87 | 1.43 | 2.73 | 0.0007 | 0.1922 | 0.0030 |
| K7TNN3 | **Peroxisome biogenesis protein 6-like** | atTSGR | T(2): 99.9 | 0.46 | 1.34 | 0.54 | 0.0002 | 0.2843 | 0.0483 |
| K7UBY5 | **Heterogeneous nuclear ribonucleoprotein r-like** | gsRDDSEEPEEDDDNDER | S(2): 82.0; S(6): 82.0 | 0.63 | 0.84 | 0.47 | 0.0231 | 0.5628 | 0.0147 |
| K7UDL1 | **Hypothetical protein ZEAMMB73_520864** | sEVNDEDDEEGsEEDEDDDE | S(12): 100.0 | 0.57 | 0.61 | 0.45 | 0.0062 | 0.0894 | 0.0099 |
| K7V1I2 | **Arginine serine-rich protein 45-like** | gsPSPR | S(2): 100.0 | 2.72 | 1.33 | 2.12 | 0.0012 | 0.2974 | 0.0267 |
| K7VQH0 | **Bzip transcription factor superfamily protein** | eGGVEsDEEIR | S(6): 100.0 | 1.50 | 1.33 | 2.07 | 0.1960 | 0.2901 | 0.0327 |
| Q41735 | **G-box binding factor 1** | aAAsPSsPR | S(7): 98.5 | 1.61 | 1.46 | 2.84 | 0.1281 | 0.1688 | 0.0021 |
| Q5QJA2 | **Harpin binding protein 1** | gLAAsQEDLDR | S(5): 100.0 | 1.58 | 1.25 | 2.03 | 0.1414 | 0.4040 | 0.0373 |

**Note: CK**: control; **D**: drought stress; **H**: heat stress; **DH**: combined drought and heat stress.
