# Supplementary material for: Phosphoproteomic analysis of the response of maize leaves to drought, heat and their combination stress
Source: Front Plant Sci. 2015 May 5;6:298. doi: 10.3389/fpls.2015.00298 (PMC4419667; doi:10.3389/fpls.2015.00298)
Supplement: Supplementary file 3 [file Table3.DOC]

**Table S3︱The P**roteins with significant phosphorylation level changes only under D and H stress.

| **Protein Group Accessions** | **Protein name** | **Sequence** | **PhosphoRS Site Probabilities (>75%)** | **Ratio of phosphorylation level** | | | **P-Value** | | |
| --- | --- | --- | --- | --- | --- | --- | --- | --- | --- |
|  |  |  |  | D/CK | H/CK | DH/CK | D/CK | H/CK | DH/CK |
| B4FBM8 | **RNA-binding protein 39-like isoform x2** | nLVQsNATSGGAASGGAR | S(5): 71.4 | 0.24 | 0.30 | 0.65 | 0.0000 | 0.0000 | 0.1644 |
| B4FN85 | **Mediator of RNA polymerase ii transcription subunit 6-like** | lEkIGNVETEPDAVAsESk | S(16): 93.3 | 0.66 | 0.56 | 0.56 | 0.0399 | 0.0451 | 0.0592 |
| B6SZ69 | **Heat shock cognate 70 kDa protein 2** | mYQGAAGPDmAGGmDQDAsPAGGSGAGPk | Y(2): 90.2 | 2.11 | 0.33 | 1.46 | 0.0159 | 0.0001 | 0.2708 |
| B6T1H0 | **TPA: 40s ribosomal protein s9** | asAAtSA | S(2): 100.0; T(5): 99.9 | 2.35 | 0.31 | 0.93 | 0.0059 | 0.0000 | 0.8160 |
| B6U7Q4 | **Protein notum homolog** | gGGGAGGPtTmR | T(9): 50.0; T(10): 50.0 | 1.96 | 1.80 | 1.40 | 0.0295 | 0.0365 | 0.3193 |
| B6UDS8 | **Uncharacterized protein LOC100278798** | hWASGARsR | S(8): 99.7 | 0.50 | 0.44 | 0.71 | 0.0008 | 0.0043 | 0.2587 |
| B7ZYR5 | **TPA: leucine-rich repeat receptor-like protein kinase family protein** | eDLGGGASGsAAAVAAAAAGGAAGEQSR | S(10): 80.0 | 0.57 | 0.25 | 0.93 | 0.0067 | 0.0000 | 0.8133 |
| B7ZZ27 | **SPF1-like DNA-binding protein** | dGNSSAFDQNEQSNDTTSGLsGAk | S(21): 78.8 | 4.16 | 3.22 | 1.45 | 0.0000 | 0.0000 | 0.2744 |
| C0PBP2 | **Transcription initiation factor alpha subunit** | aAGLNEsDADEDEEDEDQEDDSSPVLAPk | S(7): 100.0 | 0.36 | 0.49 | 1.00 | 0.0000 | 0.0121 | 0.9977 |
| C0PF35 | **Unknown** | dsSQGR | S(2): 80.0 | 0.46 | 0.26 |  | 0.0002 | 0.0000 | 1.0000 |
| C0PND4 | **Receptor-like protein kinase herk 1-like** | vSsLDISSTDQSR | S(3): 97.9 | 2.20 | 1.67 | 1.47 | 0.0107 | 0.0648 | 0.2545 |
| K7UB62 | **Uncharacterized protein** | tMTGLASSTDQSsAsSSPR | S(13): 81.1; S(15): 81.1 | 1.96 | 1.61 | 1.28 | 0.0305 | 0.0850 | 0.4769 |
| K7V664 | **Ring-h2 finger protein atl13-like** | dLAAGDGtGSGNSSGR | T(8): 82.9 | 0.62 | 0.55 | 0.94 | 0.0200 | 0.0374 | 0.8240 |
| K7VD18 | **Hypothetical protein ZEAMMB73_026023** | tSDADsEAGSGSGGGGR | S(6): 93.5 | 3.80 | 3.28 | 1.13 | 0.0000 | 0.0000 | 0.7227 |
| K7W2Z7 | **Protein kinase superfamily protein** | rPsYSLsQNQNQAPPAAR | S(3): 77.0; S(7): 77.0 | 2.04 | 2.59 | 1.44 | 0.0216 | 0.0008 | 0.2825 |

**Note: CK**: control; **D**: drought stress; **H**: heat stress; **DH**: combined drought and heat stress.
