# Supplementary material for: Phosphoproteomic analysis of the response of maize leaves to drought, heat and their combination stress
Source: Front Plant Sci. 2015 May 5;6:298. doi: 10.3389/fpls.2015.00298 (PMC4419667; doi:10.3389/fpls.2015.00298)
Supplement: Supplementary file 4 [file Table4.DOC]

**Table S4︱The P**roteins with significant phosphorylation level changes only under D stress.

| **Protein Group Accessions** | **Protein name** | **Sequence** | **PhosphoRS Site Probabilities** (>75%) | **Ratio of phosphorylation level** | | | **P-Value** | | |
| --- | --- | --- | --- | --- | --- | --- | --- | --- | --- |
|  |  |  |  | D/CK | H/CK | DH/CK | D/CK | H/CK | DH/CK |
| A3KLI0 | **RAB17 protein** | sGsSSSSSSEDDGMGGR | S(3): 100.0 | 1.90 | 1.30 | 1.92 | 0.0392 | 0.4119 | 0.0522 |
| B4FAW3(B6SKI1) | **Photosystem i reaction center subunit ii** | gFVAPQLDPSTPSPIFGGStGGLLR | T(20): 77.5 | 0.63 | 0.80 | 0.55 | 0.0235 | 0.4645 | 0.0540 |
| B4FBA9 | **Zinc finger c-x8-c-x5-c-x3-h type family protein** | gANEEVsSINVDEDPNVPYER | S(7): 50.0; S(8): 50.0; Y(19): 0.1 | 0.36 | 0.69 | 0.79 | 0.0000 | 0.2117 | 0.4353 |
| B4FF32 | **Nuclear-pore anchor-like isoform x3** | aAVEPDQSPITQPGAADASPsR | S(21): 80.0 | 0.63 | 0.65 | 0.73 | 0.0515 | 0.0721 | 0.0107 |
| B4FFI4 | **2-Aminoethanethiol dioxygenase-like** | aAAAsGGPR | S(5): 100.0 | 0.59 | 0.79 | 0.72 | 0.0111 | 0.4296 | 0.2822 |
| B4FJG1 | **Chlorophyll a-b binding protein chloroplastic-like** | nEAGGIIGtRFESSDVk | T(9): 100.0 | 2.64 | 0.71 | 1.21 | 0.0017 | 0.2370 | 0.5760 |
| B4FNM4 | **60s Acidic ribosomal protein p0** | aAEPEEEsDEEMGFSLFDD | S(8): 100.0 | 0.60 | 0.76 | 0.82 | 0.3116 | 0.7738 | 0.6314 |
| B4FQM0 | **ZF-HD homeobox protein** | vQPSTASGGsPQsS | S(10): 100.0; S(13): 97.8 | 0.64 | 1.02 | 1.06 | 0.0315 | 0.9087 | 0.8615 |
| B4FR07 | **Chaperone protein dnaj 15-like** | yTIEQsIPEsPENGSVDGR | S(10): 99.6 | 0.44 | 1.00 | 0.76 | 0.0001 | 0.9636 | 0.3632 |
| B4FRE3 | **Ankyrin repeat domain-containing protein 2-like** | tVVsPR | S(4): 100.0 | 2.13 | 0.85 | 0.89 | 0.0147 | 0.6064 | 0.7033 |
| B4FRX1;B6TB14 | **Splicing arginine serine-rich 7** | sRSPQDQVmsPPPk | S(3): 93.9; S(10): 100.0 | 0.65 | 1.03 | 0.70 | 0.0392 | 0.8765 | 0.2511 |
| B4FS10 | **TPA: hypothetical protein ZEAMMB73_767959** | aktTAGk | T(3): 99.9 | 0.67 | 1.04 | 0.94 | 0.0491 | 0.8579 | 0.8454 |
| B4FTL9 | **Bidirectional sugar transporter sweet13-like** | lsPALPER | S(2): 100.0 | 0.60 | 0.86 | 0.88 | 0.0133 | 0.6418 | 0.6659 |
| B4FXH4 | **Villin-2-like isoform 1** | iVITPAGPSGPSsPQSEAGESNVFHQEk | S(13): 82.7 | 0.50 | 0.90 | 0.68 | 0.0009 | 0.7376 | 0.2095 |
| B4FXM8 | **Nuclear-interacting partner of alk** | dTELDEASNETQPEtGSPLRk | T(15): 80.0 | 0.53 | 0.83 | 0.72 | 0.0020 | 0.5427 | 0.2944 |
| B4FXM8 | **Nuclear-interacting partner of alk** | dTELDEASNETQPETGsPLR | S(17): 99.9 | 2.06 | 0.90 | 0.84 | 0.0194 | 0.7557 | 0.5658 |
| B4FZB5 | **Uncharacterized protein LOC100274151** | nGYtSVLEAR | Y(3): 33.3; T(4): 33.3; S(5): 33.3 | 0.25 | 0.89 | 0.66 | 0.0000 | 0.7075 | 0.1814 |
| B4FZE7 | **Pleckstriny domain-containing expressed** | eNDLGISNQGsPDQVFSR | S(11): 100.0 | 0.60 | 0.73 | 0.63 | 0.0127 | 0.2922 | 0.1360 |
| B4G137 | **Ankyrin repeat domain-containing protein 2-like** | sEGSsDER | S(5): 98.1 | 1.98 | 0.76 | 1.27 | 0.0274 | 0.3605 | 0.4783 |
| B6SJ48 | **Hypothetical protein** | ePVsPGtPSSVAAGR | S(4): 100.0; | 2.41 | 0.89 | 1.13 | 0.0045 | 0.7286 | 0.7266 |
| B6SL90 | **Splicing arginine serine-rich 7** | eEsPYDNGYR | S(3): 100.0 | 1.91 | 1.07 | 1.17 | 0.0375 | 0.7634 | 0.6454 |
| B6SSC1 | **Lipid phosphate phosphatase 3** | dtSPILESMEsGR | S(11): 99.9 | 0.46 | 0.96 | 1.20 | 0.0002 | 0.9415 | 0.6013 |
| B6ST41 | **RPT2-like protein** | gGRStPPsSPSR | S(4): 97.3 | 0.56 | 1.05 | 0.79 | 0.0046 | 0.8189 | 0.4378 |
| B6SVK8 | **Protein serine threonine kinase** | aAQGSLDsPTGGG | S(8): 99.7 | 0.64 | 0.68 | 0.89 | 0.0292 | 0.1898 | 0.7114 |
| B6SWP2 | **RAC gtpase activating protein 1** | yLANADSDVLQGsGSPER | S(13): 76.0 | 0.67 | 0.98 | 0.91 | 0.0499 | 0.9767 | 0.7516 |
| **B6SXN6** | **Bel1-type homeodomain protein** | gGASSsGAAAQsPSSAPNkEPPQLSPADR | S(6): 77.1 | 2.18 | 0.74 | 0.78 | 0.0120 | 0.3104 | 0.4115 |
| B6T6V5 | **Ubiquitin carboxyl-terminal hydrolase 6-like** | sALLSYsDTVR | S(7): 75.0 | 0.65 | 0.86 | 0.78 | 0.0333 | 0.6300 | 0.4234 |
| B6T8F4 | **RAN-binding protein 1** | sEASEEEDASAAAAAGEEEDTGAQVAPIVR | S(1): 75.2 | 0.41 | 0.71 | 0.76 | 0.0000 | 0.2451 | 0.3836 |
| B6TAW2 | **Chaperone protein dnaj 10** | nIDNmDGNDGsSPDSSPNR | S(11): 95.6 | 0.56 | 0.89 | 1.04 | 0.0046 | 0.7286 | 0.9148 |
| B6TCM5 | **DUF1664 domain family protein isoform 1** | hNmANAVsSMtkHLEQVQssLAAAk | T(11): 94.3; S(19): 94.3; S(20): 94.3 | 0.47 | 1.05 | 0.90 | 0.0003 | 0.8240 | 0.7409 |
| B6TNS0 | **Nucleolar protein 56-like** | eAsEEAEPk | S(3): 100.0 | 0.62 | 0.77 | 0.85 | 0.0213 | 0.3779 | 0.6076 |
| B6TV01 | **Calmodulin-related protein touch-induced** | isPSELAAVSR | S(2): 99.9 | 1.90 | 0.92 | 1.22 | 0.0384 | 0.8039 | 0.5579 |
| B6U3H3 | **Calvin cycle protein cp12- chloroplastic-like** | ssGPATPPEISDk | S(2): 96.2 | 0.59 | 1.60 | 1.49 | 0.0113 | 0.0911 | 0.2391 |
| B6U6U2 | **Hexose transporter** | eGEDYAsDHGGDDIEDNLQSPLISR | S(7): 98.0 | 1.87 | 0.69 |  | 0.0430 | 0.2098 | 1.0000 |
| B6U6Y9 | **ATP binding protein** | ltFcVEGNIsVGk | T(2): 100.0; S(10): 100.0 | 0.55 | 0.78 | 0.55 | 0.0035 | 0.4164 | 0.0518 |
| B6UCP3 | **Ferric-chelate reductase 1-like** | lLITPtHGDSDSEQsYk | T(6): 86.8 | 0.65 | 1.27 | 1.54 | 0.0365 | 0.3678 | 0.2052 |
| B6UF28 | **Hypothetical protein** | nEAAsR | S(5): 100.0 | 0.60 | 0.90 | 0.87 | 0.0124 | 0.7617 | 0.6606 |
| B6UHG9 | **Uncharacterized abhydrolase domain-containing protein ddb_g0269086-like** | gGVPYGGPGATYGGsPSFATGGGQQPFR | S(15): 75.5 | 0.59 | 0.63 | 0.71 | 0.0101 | 0.1094 | 0.2723 |
| B6UHZ8 | **SSXT protein** | sTGGDDGGsD | S(9): 100.0 | 0.58 | 0.88 | 0.82 | 0.0075 | 0.6955 | 0.5145 |
| B7ZZU5 | **TPA: heterogeneous nuclear ribonucleoprotein r** | dDTGsSPTAk | S(5): 79.4 | 0.62 | 0.93 | 0.77 | 0.0208 | 0.8430 | 0.3975 |
| B8A1D5 | **Glycerol 3-phosphate permease** | dVADAQGtYYSDEV | T(8): 82.9 | 0.55 | 0.64 | 0.86 | 0.0033 | 0.1266 | 0.6155 |
| C0HF02 | **Chlorophyll a-b binding protein chloroplastic-like** | nEAGGIIGtRFESSEVk | T(9): 100.0 | 2.26 | 0.73 | 1.34 | 0.0086 | 0.2768 | 0.3884 |
| B4FJG1 | **Chlorophyll a-b binding protein chloroplastic-like** | nEAGGIIGtR | T(9): 100.0 | 2.58 | 0.58 | 0.95 | 0.0022 | 0.0570 | 0.8747 |
| C0HIN5 | **Arginine serine-rich splicing factor rs2z37a transcript i** | vNNGDDPsPk | S(8): 100.0 | 1.92 | 1.26 | 1.45 | 0.0361 | 0.3947 | 0.2717 |
| C0P3W9 | **Phosphoenolpyruvate carboxykinase** | gEAAAQGAPstPR | S(10): 100.0; T(11): 100.0 | 0.64 | 0.59 | 0.61 | 0.0292 | 0.0664 | 0.1062 |
| C0P3W9 | **Phosphoenolpyruvate carboxykinase** | gGAHsPFAVAISEEER | S(5): 100.0 | 0.64 | 0.87 | 0.81 | 0.0286 | 0.6626 | 0.4843 |
| C0P9H7 | **Arginine serine-rich splicing factor rsp41** | rsPsPYGR | S(2): 100.0; S(4): 99.9 | 1.90 | 0.82 | 0.97 | 0.0391 | 0.5059 | 0.9038 |
| C0PD11 | **Mitochondrial glycoprotein** | aFSLTAAGtHAtPR | T(9): 76.6; T(12): 76.6 | 0.40 | 0.74 | 0.68 | 0.0000 | 0.3058 | 0.2129 |
| C0PE12 | **Hypothetical protein** | aQIAtVR | T(5): 100.0 | 0.57 | 0.98 | 0.78 | 0.0067 | 0.9972 | 0.4281 |
| C0PE12 | **Hypothetical protein** | aQAEEEtLASER | T(7): 100.0 | 0.55 | 0.95 | 1.06 | 0.0043 | 0.9118 | 0.8791 |
| C0PNW3 | **Magnesium transporter mrs2-b-like isoform x1** | aYPSQVAsASSPALPSAPPGSAGR | S(8): 82.8 | 0.40 | 1.08 | 0.85 | 0.0000 | 0.7560 | 0.5971 |
| C4J038 | **Calcium-dependent protein kinase** | aTAPDsGR | S(6): 100.0 | 2.44 | 0.72 | 0.70 | 0.0039 | 0.2596 | 0.2511 |
| C4JAN4 | **Protein phosphatase 2c family protein** | sIsAEGLR | S(3): 100.0 | 0.65 | 0.58 | 0.60 | 0.0385 | 0.0620 | 0.0983 |
| K7TMH1 | **Hypothetical protein ZEAMMB73_634657** | lVTASPsLR | S(7): 100.0 | 0.64 | 1.26 | 1.33 | 0.0327 | 0.3916 | 0.3994 |
| K7TSC5 | **C3h11 c3h type transcription partial** | sAsFIAsPR | S(3): 100.0; S(7): 100.0 | 1.94 | 0.96 | 1.09 | 0.0324 | 0.9296 | 0.8143 |
| K7U162 | **RNA polymerase ii-associated factor 1 homolog** | vEDIDQYSEEYsE | S(12): 97.9 | 0.39 | 1.00 | 0.75 | 0.0000 | 0.9442 | 0.3454 |
| K7U2X9 | **Geranylgeranyl pyrophosphate synthase 4** | sAsGNGk | S(3): 100.0 | 2.00 | 0.89 | 1.19 | 0.0258 | 0.7316 | 0.6100 |
| K7U9T7 | **Protein phosphatase 2c and cyclic nucleotide-binding kinase domain-containing isoform x1** | vTLQPSQQVVGLAGPEsPLIVSSNTNNQR | S(17): 100.0 | 0.44 | 1.07 | 0.83 | 0.0001 | 0.7758 | 0.5529 |
| K7UE26 | **Pollen-specific protein sf3** | tSQAGsLEEDEQEY | S(6): 99.8 | 1.84 | 1.13 | 1.17 | 0.0487 | 0.6394 | 0.6527 |
| K7UHH6 | **Zinc finger c-x8-c-x5-c-x3-h type family protein** | vGDYDDEGNIVsSSNIFADR | S(12): 83.3 | 0.22 | 0.58 | 0.68 | 0.0000 | 0.0611 | 0.2164 |
| K7UQX6 | **Disease resistance protein rpp13** | vLtcRMyDLVHDFIVsk | T(3): 100.0; Y(7): 100.0; S(16): 100.0 | 0.20 | 0.77 | 0.73 | 0.0000 | 0.3906 | 0.3173 |
| K7UTD5 |  | sSGGAsPR | S(6): 100.0 | 2.06 | 1.23 | 1.42 | 0.0201 | 0.4297 | 0.3073 |
| K7UTW7 | **Hypothetical protein ZEAMMB73_512164** | eAGDLVAGGcGEEGsPQADAk | S(15): 100.0 | 0.61 | 0.57 | 0.93 | 0.0155 | 0.0501 | 0.7999 |
| K7UVT7 | **Skin secretory protein xp2-like** | sSEESEAANDDDDAsA | S(15): 100.0 | 0.63 | 0.80 | 0.80 | 0.0226 | 0.4509 | 0.4744 |
| K7V1I2 | **Arginine serine-rich protein 45-like** | gRsPsPPPk | S(3): 100.0; S(5): 100.0 | 2.44 | 0.96 | 0.98 | 0.0040 | 0.9207 | 0.9484 |
| K7V5J0 | **ABC transporter f family member 4-like** | lGsDDDsENDR | S(3): 100.0; S(7): 100.0 | 2.14 | 1.44 | 1.36 | 0.0139 | 0.1817 | 0.3718 |
| K7VM50 | **Perq amino acid-rich with gyf domain-containing protein 2** | qGssASLAGDDTNFSEEAVSk | S(3): 80.0; S(4): 80.0 | 0.52 | 0.93 | 0.72 | 0.0016 | 0.8460 | 0.2863 |
| K7VUD1 | **Hypothetical protein ZEAMMB73_259781** | lQHARAsSAPHPSR | S(7): 83.3 | 1.97 | 1.21 | 0.93 | 0.0289 | 0.4688 | 0.8214 |
| M1H541 | **Splicing arginine serine-rich 2** | sYNHsPsPPR | S(5): 99.5; S(7): 100.0 | 0.53 | 0.70 | 0.61 | 0.0021 | 0.2310 | 0.1144 |
| P27789 | **Ferredoxin- chloroplastic precursor** | atVLSSPR | T(2): 100.0 | 2.62 | 0.92 | 0.94 | 0.0018 | 0.8009 | 0.8454 |
| Q41812 | **Phd-finger family homeodomain protein** | sAsNNk | S(3): 100.0 | 2.27 | 0.89 | 1.28 | 0.0082 | 0.7075 | 0.4655 |
| Q84UX7 | **Global transcription factor group e** | vDDEEDDEQcsLH | S(11): 100.0 | 0.52 | 1.29 | 0.94 | 0.0014 | 0.3438 | 0.8267 |
| Q8H6B2 | **Bromodomain protein 103** | fsALSyGsSIPLVyRPR | Y(14): 99.5 | 1.96 | 0.71 | 0.64 | 0.0304 | 0.2370 | 0.1456 |
| Q8L8G5 | **Nucleosome chromatin assembly factor group a** | eSAAEtPEEQk | T(6): 100.0 | 0.55 | 1.07 | 1.00 | 0.0040 | 0.7708 | 0.9977 |
| Q8W149 | **Cell division cycle 5-like** | eIQTPNPmATPLASPGPGItPR | S(14): 96.7 | 0.43 | 0.95 | 1.22 | 0.0001 | 0.8940 | 0.5661 |

**Note: CK**: control; **D**: drought stress; **H**: heat stress; **DH**: combined drought and heat stress.
