# Supplementary material for: Phosphoproteomic analysis of the response of maize leaves to drought, heat and their combination stress
Source: Front Plant Sci. 2015 May 5;6:298. doi: 10.3389/fpls.2015.00298 (PMC4419667; doi:10.3389/fpls.2015.00298)
Supplement: Supplementary file 5 [file Table5.DOC]

**Table S5︱The Proteins with significant phosphorylation level changes only under H stress.**

| **Protein Group Accessions** | **Protein name** | **Sequence** | **PhosphoRS Site Probabilities**  (>75%) | **Ratio of phosphorylation level** | | | **P-Value** | | |
| --- | --- | --- | --- | --- | --- | --- | --- | --- | --- |
|  |  |  |  | D/CK | H/CK | DH/CK | D/CK | H/CK | DH/CK |
| B4F7Z5 | **Splicing arginine serine-rich 7 isoform 1** | dEsPYANDA | S(3): 100.0 | 0.92 | 0.50 | 0.74 | 0.6829 | 0.0167 | 0.3280 |
| B4FQK5 | **Eukaryotic peptide chain release factor subunit 1-1** | sFDELsDDDDVYEDsD | S(6): 100.0; S(15): 100.0 | 0.80 | 0.56 | 0.79 | 0.2735 | 1.0000 | 0.0477 |
| B4FRV0 | **DNA-binding protein** | tPsSGDLk | S(3): 98.1 | 0.89 | 0.55 | 0.74 | 0.5670 | 0.0380 | 0.3194 |
| B6SJ48 | **Hypothetical protein** | ePVsPGTPSSVAAGR | S(4): 100.0 | 0.82 | 0.53 | 0.59 | 0.3173 | 0.0293 | 0.0898 |
| B6T090 | **Thioredoxin h-type** | hAAPAPASAsA | S(10): 100.0 | 1.08 | 0.57 | 0.73 | 0.8080 | 0.0493 | 0.3068 |
| B6TI42 | **At-hook protein 1** | qQQQQQLAPSPAPLNLAPTGVAAGPsSPPSR | S(26): 49.4 | 1.00 | 1.81 | 1.22 | 0.9962 | 0.0343 | 0.5579 |
| B6TJW1 | **Hypothetical protein** | eGSQLEPDGsSAR | S(10): 80.0 | 1.15 | 0.22 | 1.07 | 0.6716 | 0.0000 | 0.8441 |
| B6TNC1 | **Poly -specific endoribonuclease-b-like** | aAQSsDPDDDGEEERDER | S(5): 97.0 | 0.88 | 0.56 | 0.56 | 0.5061 | 0.0465 | 0.0600 |
| B6TT68 | **F-box domain containing protein** | gVDADDPAxsTcTDGDGDAVGR | S(10): 78.6 | 0.76 | 0.38 | 0.59 | 0.1860 | 0.0008 | 0.0898 |
| B6TWG6 | **Cop9 signalosome complex subunit 6a** | aQAAcSGDSsSPSSSAPGAGQPPR | S(10): 93.8 | 0.95 | 0.52 | 0.77 | 0.7754 | 0.0229 | 0.3882 |
| B6U787 | **Acetylglutamate kinase** | isAtSTAAPSPSSAAAATASLsR | S(22): 83.2 | 1.18 | 1.84 | 0.92 | 0.5999 | 0.0293 | 0.7812 |
| B6U8S7 | **Carbohydrate transporter sugar porter transporter** | gGGsPVAAVQDASDDGAR | S(4): 100.0 | 0.84 | 1.82 | 1.83 | 0.3737 | 0.0321 | 0.0751 |
| C0HE50 | **Zinc finger (c3hc4-type ring finger)** | vTIScsPSsTR | S(9): 80.0 | 1.60 | 0.54 | 0.61 | 0.1291 | 0.0334 | 0.1132 |
| C0HIQ2 | **Something about silencing protein 10-like isoform x4** | nAYYAPGEQsGDDEVDYEEAQR | S(10): 99.9 | 0.79 | 0.40 | 0.79 | 0.2580 | 0.0012 | 0.4353 |
| C0P9L7 | **E3 ubiquitin-protein ligase rglg2-like isoform x1** | sSsFDQQTSGASQQR | S(3): 97.4 | 1.34 | 0.55 | 0.66 | 0.3547 | 0.0380 | 0.1814 |
| C0PCR0 | **Dhhc-type zinc finger domain-containing protein** | vAHAsPDLSR | S(5): 100.0 | 0.88 | 0.57 | 0.86 | 0.5238 | 0.0493 | 0.6261 |
| C0PD66 | **Uncharacterized protein LOC100383120** | sVESGGGQPDtPPTtPQR | T(11): 100.0; T(15): 97.1 | 0.91 | 1.86 | 1.38 | 0.6411 | 0.0268 | 0.3414 |
| C0PDN0 | **TPA: trehalose phosphatase synthase family protein** | qGsFGLR | S(3): 100.0 | 1.57 | 1.83 | 1.91 | 0.1447 | 0.0308 | 0.0569 |
| C4J1T9 | **Web family protein at5g55860-like** | tAEAQPSAGAEELGsGSPPVPQTSAGk | T(1): 75.0; S(15):75.0 | 1.20 | 2.05 | 1.11 | 0.5570 | 0.0108 | 0.7625 |
| C4J4G0 | **Uncharacterized loc100501590** | qSsLAPER | S(3): 100.0 | 0.94 | 0.57 | 0.64 | 0.7405 | 0.0493 | 0.1456 |
| C4J9U8 | **Poly polymerase-like isoform x2** | nsPQSGSTGDETR | S(2): 99.7 |  | 1.93 | 0.62 | 1.0000 | 0.0196 | 0.1205 |
| K7UDH3 | **Protein stichel-like** | sQDGmDLSVHcADLHEsDPR | S(8): 92.6 | 0.83 | 0.32 | 1.01 | 0.3645 | 0.0001 | 0.9813 |
| K7UTP6 | **nitrate reductase apoenzyme** | lDDAsDDEDEEQEDWR | S(5): 100.0 | 1.47 | 0.32 | 0.69 | 0.2164 | 0.0001 | 0.2307 |
| K7V5E8 | **Translocase of chloroplast chloroplastic-like isoform x1** | dADGDDsLGGGEASEESANk | S(7): 100.0 | 0.89 | 0.56 | 0.63 | 0.5670 | 0.0465 | 0.1373 |
| K7V7V5 | **Motile sperm domain-containing protein 2-like** | mAtVmAAAASAVEAPATAk | T(3): 50.0; S(10): 50.0; T(17): 0.0 | 1.06 | 0.51 | 0.72 | 0.8673 | 0.0194 | 0.2903 |
| K7V8I9 | **Hypothetical protein ZEAMMB73_938746** | yEASGDEsDNDGNTNS | S(8): 97.9 | 0.93 | 0.45 | 0.82 | 0.7251 | 0.0054 | 0.5170 |
| K7VEK3 | **Proteasome subunit beta type-5-a-like** | tPVEQEMADAPTAsA | S(14): 99.9 | 1.02 | 0.55 | 0.75 | 0.9718 | 0.0392 | 0.3609 |
| K7VTF9 | **DUF1296 domain containing family protein** | nPsDSNLSGAAQANQmWPNSY | S(3): 77.9 | 1.73 | 1.90 | 1.33 | 0.0787 | 0.0220 | 0.4019 |
| K7WC16 | **Uncharacterized protein** | vLsPEGDVDEDTVR | S(3): 100.0 | 1.13 | 1.83 | 1.44 | 0.7010 | 0.0312 | 0.2816 |
| Q94IQ8 | **Methyl- binding domain containing expressed** | dAETGQDAPsEDGTk | S(10): 99.9 | 0.71 | 0.54 | 0.64 | 0.0953 | 0.0303 | 0.1526 |

**Note: CK**: control; **D**: drought stress; **H**: heat stress; **DH**: combined drought and heat stress.
