# Supplementary material for: Phosphoproteomic analysis of the response of maize leaves to drought, heat and their combination stress
Source: Front Plant Sci. 2015 May 5;6:298. doi: 10.3389/fpls.2015.00298 (PMC4419667; doi:10.3389/fpls.2015.00298)
Supplement: Supplementary file 6 [file Table6.DOC]

**Table S6︱The proteins with significant phosphorylation level changes only under DH stress.**

| **Protein Group Accessions** | **Protein name** | **Sequence** | **PhosphoRS Site Probabilities**  (>75%) | **Ratio of phosphorylation level** | | | **P-Value** | | |
| --- | --- | --- | --- | --- | --- | --- | --- | --- | --- |
|  |  |  |  | D/CK | H/CK | DH/CK | D/CK | H/CK | DH/CK |
| B4F8R0 | **TPA: protein phosphatase 2c family protein** | gNcVASGGtTtVTAAGAAGEDGRR | T(11): 79.4 | 1.07 | 0.80 | 0.42 | 0.8409 | 0.4727 | 0.0051 |
| B4FC96 | **ZN-containing protein** | gAcGAPRPsPsPSPR | S(9): 100.0; S(11): 99.5 | 0.78 | 0.61 | 0.49 | 0.2122 | 0.0872 | 0.0212 |
| B4FQK5 | **Eukaryotic peptide chain release factor subunit 1-1** | sFDELsDDDDVYEDSD | S(1): 0.0; S(6): 99.8; Y(12): 0.1; S(15): 0.1 | 0.80 |  | 0.54 | 0.2761 | 0.0437 | 0.4402 |
| B4FXQ9 | **Coiled-coil domain containing 55** | eSsIESER | S(3): 100.0 | 0.92 | 0.63 | 0.43 | 0.6867 | 0.1082 | 0.0063 |
| B4FZ13 | **Unknown** | nNENAPQEYQAAsEIIPDk | S(13): 100.0 | 0.77 | 0.68 | 0.48 | 0.1924 | 0.1951 | 0.0174 |
| B4G1A9 | **BSD domain containing protein** | aSDPTSAETGESDEQPDtPSR | T(18): 97.5 | 0.83 | 0.69 | 2.04 | 0.3584 | 0.1969 | 0.0356 |
| B6T992 | **UPF0690 protein c1orf52 homolog isoform x1** | aAsTAEAR | S(3): 50.0; T(4): 50.0 | 1.29 | 1.41 | 2.03 | 0.4113 | 0.2148 | 0.0370 |
| B6TG30 | **30s ribosomal protein chloroplastic-like** | sQLLPPLQsDDDQEPSSRE | S(9): 100.0 | 0.95 | 1.16 | 1.95 | 0.7715 | 0.5693 | 0.0487 |
| B6TG30 | **30s ribosomal protein chloroplastic-like** | sQLLPPLQsDDDQEPSSR | S(9): 100.0 | 1.11 | 1.39 | 2.17 | 0.7533 | 0.2251 | 0.0227 |
| B6TVL4 | **Calcium sensing receptor** | iGtASSAsR | T(3): 99.9 | 1.22 | 1.05 | 2.10 | 0.5234 | 0.8163 | 0.0286 |
| B6TY90 | **Gibberellin receptor gid1l2** | csAGVDEATGVTSk | S(2): 78.3 | 1.39 | 1.23 | 2.04 | 0.2950 | 0.4379 | 0.0358 |
| B6U899 | **Histone-lysine n-methyltransferase family protein** | ssISLDDDDDEPYSGNQGLASER | S(1): 84.7; S(2): 84.7 | 0.74 | 0.97 | 0.54 | 0.1400 | 0.9562 | 0.0483 |
| B8A326 | **Plasma membrane h+-transporting atpase-like protein** | gLDIDtIQQNYtV | T(6): 99.8; T(12): 96.5 | 1.32 | 0.82 | 2.45 | 0.3795 | 0.5087 | 0.0083 |
| C0P6V3 | **TPA: hmg1 protein** | skSEVEDDEQDGNEDEDE | S(1): 50.0; S(3): 50.0 | 0.87 | 0.57 | 0.53 | 0.5026 | 0.0501 | 0.0432 |
| C0PM56 | **Chloroplast post-illumination chlorophyll fluorescence increase protein** | lDIVSGcTDPSSDmFDPLATVDDGScPLEsDSEE | S(30): 75.0 | 1.45 | 1.20 | 2.21 | 0.2378 | 0.4921 | 0.0194 |
| C4J2C3 | **Unknown** | qGIsmsFcLAVPR | S(4): 100.0; S(6): 100.0 | 1.07 | 0.85 | 0.47 | 0.8481 | 0.5947 | 0.0156 |
| K7TLV1 | **Polyadenylate-binding protein 2** | dAALsPAAQIGTLTSALANANPEQQR | S(5): 100.0 | 0.74 | 1.01 | 0.47 | 0.1435 | 0.9387 | 0.0156 |
| K7TUM2 | **Eukaryotic translation initiation factor isoform 4g-1-like** | tSsINTR | S(3): 98.2 | 1.45 | 1.44 | 2.06 | 0.2325 | 0.1881 | 0.0340 |
| K7U0Z2 | **Pumilio homolog 1-like isoform x1** | sGSTsPSLVR | S(1): 100.0; S(3): 94.2 | 0.77 | 0.72 | 0.53 | 0.1989 | 0.2660 | 0.0390 |
| K7UY68 | **Cmo protein** | sLAGVYsARATRPMPLRAGAR | S(1): 75.6; S(7): 75.6 | 1.37 | 0.82 | 0.36 | 0.3116 | 0.5285 | 0.0009 |
| K7V792 | **Splicing factor 3b subunit 1-like isoform x1** | vGDAtPsVR | T(5): 100.0; S(7): 100.0 | 0.70 | 0.73 | 0.50 | 0.0863 | 0.2900 | 0.0235 |
| K7VZG4 | **Hypothetical protein ZEAMMB73_894229** | sNtSSAPR | S(1): 86.3; T(3): 86.3 |  |  | 0.53 | 1.0000 | 1.0000 | 0.0378 |
| P49106 | **14-3-3-like protein gf14-6** | rDsSEGQ | S(3): 100.0 | 1.22 | 0.78 | 0.53 | 0.5268 | 0.4086 | 0.0396 |

**Note: CK**: control; **D**: drought stress; **H**: heat stress; **DH**: combined drought and heat stress.
