# Supplementary material for: Phosphoproteomic analysis of the response of maize leaves to drought, heat and their combination stress
Source: Front Plant Sci. 2015 May 5;6:298. doi: 10.3389/fpls.2015.00298 (PMC4419667; doi:10.3389/fpls.2015.00298)
Supplement: Supplementary file 7 [file Table7.DOC]

**Table S7︱Maize proteins corresponding to rice proteins in network of protein interaction under D stress.**

| **Maize query sequence** | **Rice query sequence** | **STRING protein** | **Identity** | **Bitscore** |
| --- | --- | --- | --- | --- |
| FER5 | 4344439 | 2Fe-2S iron-sulfur cluster binding domain containing protein, expressed | 75% | 196 |
| B4F7W7 | 4346661 | Myb-like DNA-binding domain containing protein, putative, expressed | 85% | 496 |
| B4FAB7 | 4327739 | CBS domain-containing protein, putative, expressed | 82% | 637 |
| B4FAW3 | 4346326 | Photosystem I reaction center subunit II, chloroplast precursor, putative, expressed | 82% | 323 |
| B4FBM8 | 4348719 | RNA recognition motif containing protein, putative, expressed | 80% | 475 |
| B4FC96 | 4343269 | Zinc finger family protein, putative, expressed | 76% | 376 |
| B4FFI8 | 4330742 | Membrane-anchored ubiquitin-fold protein, putative, expressed; | 90% | 192 |
| B4FGQ4 | 4326523 | Protein kinase APK1B, chloroplast precursor, putative, expressed | 90% | 571 |
| B4FJG1 | 4343583 | Chlorophyll A-B binding protein, putative, expressed | 90% | 494 |
| B4FKM0 | 4349004 | Oxidoreductase, short chain dehydrogenase/reductase family domain containing family, expressed; | 84% | 671 |
| B4FN85 | 4340496 | Mediator of RNA polymerase II transcription subunit 6, putative, expressed | 88% | 407 |
| B4FNM4 | 4344590 | 60S acidic ribosomal protein P0, putative, expressed; | 88% | 513 |
| B4FQU2 | 4348536 | Initiation factor 2 subunit family domain containing protein, expressed | 94% | 784 |
| B4FR07 | OsJ_01978 | Heat shock protein DnaJ, putative, expressed | 91% | 749 |
| B4FRE3 | 4334805 | Ankyrin repeat domain containing protein, expressed | 76% | 502 |
| B4FRF2 | LOC_Os01g31690.1 | Oxygen-evolving enhancer protein 1, chloroplast precursor, putative, expressed | 93% | 444 |
| B4FTL9 | 4352201 | Nodulin MtN3 family protein, putative, expressed | 78% | 409 |
| B4FY17 | 4352524 | Phospholipase C, putative, expressed | 81% | 796 |
| B4FZE7 | 4348789 | START domain containing protein, expressed | 87% | 693 |
| B4G0P6 | 4344993 | Expressed protein | 87% | 830 |
| B4G137 | 4347554 | Ankyrin repeat domain containing protein, putative, expressed | 78% | 520 |
| B4G250 | 4332357 | HSP20/alpha crystallin family protein, putative, expressed | 87% | 253 |
| B6SL90 | 4340353 | RNA recognition motif containing protein, expressed | 91% | 266 |
| B6SS20 | 4335426 | AGC_PVPK_like_kin82y.10 - ACG kinases include homologs to PKA, PKG and PKC, expressed | 77% | 1375 |
| B6SSC1 | 4345367 | Lipid phosphatase protein, putative, expressed | 89% | 566 |
| B6SVK8 | 4335687 | Serine/threonine-protein kinase NAK, putative, expressed | 85% | 726 |
| B6SWP2 | 4349806 | RhoGAP domain containing protein, expressed | 76% | 676 |
| B6SZ69 | 4332413 | DNAK family protein, putative, expressed | 94% | 1266 |
| B6T1H0 | 4333016 | 40S ribosomal protein S6, putative, expressed | 96% | 476 |
| B6T6V5 | 4326194 | Ubiquitin carboxyl-terminal hydrolase 6, putative, expressed | 93% | 910 |
| B6T890 | LOC_Os01g03500.1 | expressed protein | 84% | 481 |
| B6T8F4 | 4338501 | ranBP1 domain containing protein, expressed | 82% | 322 |
| B6TAW2 | 4346080 | Chaperone protein dnaJ 10, putative, expressed | 80% | 650 |
| B6TCM5 | 4337995 | Expressed protein | 83% | 454 |
| B6TH05 | 4349227 | Ethylene-responsive element-binding protein, putative, expressed | 85% | 419 |
| B6TNS0 | 4332851 | Nucleolar protein 5A, putative, expressed | 87% | 895 |
| B6TZS3 | 4339909 | Expressed protein | 90% | 1012 |
| B6U4K3 | 4348376 | Vacuolar-sorting receptor precursor, putative, expressed | 85% | 1070 |
| B6U6U2 | 4349251 | Transporter family protein, putative, expressed | 91% | 1346 |
| B6U6Y9 | 4338875 | Deoxynucleoside kinase family, putative, expressed | 78% | 763 |
| B6UHZ8 | 4352256 | SSXT protein, putative, expressed | 79% | 218 |
| B6UIM2 | 4324553 | 40S ribosomal protein S10, putative, expressed | 93% | 270 |
| B7ZYR5 | 4327723 | Inactive receptor kinase At2g26730 precursor, putative, expressed | 81% | 942 |
| B7ZZ27 | 4352301 | WRKY DNA-binding domain containing protein, expressed | 83% | 631 |
| B8A1D5 | 4344694 | Transporter, major facilitator family, putative, expressed | 87% | 408 |
| C0HF02 | 4343583 | Chlorophyll A-B binding protein, putative, expressed | 86% | 264 |
| C0P3W9 | 4332293 | Phosphoenolpyruvate carboxykinase, putative, expressed | 85% | 1129 |
| C0PBP2 | 4348225 | Transcription initiation factor IIF, alpha subunit domain containing protein, expressed | 88% | 897 |
| C0PD11 | 4347825 | Mitochondrial glycoprotein, putative, expressed | 77% | 289 |
| C0PD30 | 4349897 | Fructose-bisphospate aldolase isozyme, putative, expressed | 96% | 674 |
| C0PND4 | OsI_18572 | TKL_IRAK_CrRLK1L-1.10 - The CrRLK1L-1 subfamily has homology to the CrRLK1L homolog, expressed | 91% | 527 |
| C0PNW3 | 4341687 | CorA-like magnesium transporter protein, putative, expressed | 90% | 753 |
| C4J038 | 4343373 | CAMK_CAMK_like.33 - CAMK includes calcium/calmodulin depedent protein kinases, expressed; | 93% | 1001 |
| C4J1A8 | 4332374 | Protein phosphatase 2C, putative, expressed | 75% | 503 |
| C4JAN4 | 4341433 | Protein phosphatase 2C, putative, expressed | 91% | 363 |
| C4JBR4 | 4328119 | Glycine-rich protein 2, putative, expressed | 77% | 228 |
| E9NQE1 | 4328859 | Phosphoenolpyruvate carboxylase, putative, expressed | 82% | 1607 |
| F1DJV0 | 4327123 | Transcription factor HY5, putative, expressed | 77% | 183 |
| K7TSC5 | 4350492 | Zinc finger C-x8-C-x5-C-x3-H type family protein, expressed | 91% | 98.6 |
| K7U162 | 4344699 | ELF7, putative, expressed | 85% | 792 |
| K7U2X9 | 4330260 | Polyprenyl synthetase, putative, expressed | 88% | 585 |
| K7U573 | OsJ_21434 | PSP domain containing protein, expressed | 80% | 369 |
| K7U5U1 | 4329484 | DUF803 domain containing, putative, expressed | 81% | 289 |
| K7U9T7 | 4329023 | AGC_PKA/PKG_like.1 - ACG kinases include homologs to PKA, PKG and PKC, expressed | 83% | 1346 |
| K7UBY5 | 4348127 | RNA recognition motif containing protein, expressed | 77% | 594 |
| K7UKJ5 | 4330968 | RNA recognition motif containing protein, expressed | 85% | 186 |
| K7V0H0 | 4330971 | Trehalose-6-phosphate synthase, putative, expressed | 88% | 1498 |
| K7V1I2 | LOC_Os01g72890.1 | Transposon protein, putative, CACTA, En/Spm sub-class, expressed | 86% | 322 |
| K7VBI0 | 4326743 | AAA-type ATPase family protein, putative, expressed | 85% | 1382 |
| K7VD18 | 4341968 | OsSBeL1 - Putative Serine Beta-Lactamase homologue, expressed | 83% | 1600 |
| K7W2Z7 | 4331518 | Protein kinase family protein, putative, expressed | 83% | 728 |
| M1H541 | OsI_11773 | RNA recognition motif containing protein, putative, expressed | 87% | 107 |
| Q84UX7 | 4329897 | Bromodomain domain containing protein, expressed | 80% | 984 |
| Q8H6B2 | 4347572 | Bromodomain protein 103, putative, expressed | 87% | 2069 |
| Q8L8G5 | 4339460 | NAP domain containing protein, putative, expressed | 85% | 556 |
| Q8W149 | 4335542 | MYB family transcription factor, putative, expressed | 93% | 1326 |
| Q9LLI8 | 4337958 | CESA1 - cellulose synthase, expressed; | 96% | 2088 |
