# Supplementary material for: Phosphoproteomic analysis of the response of maize leaves to drought, heat and their combination stress
Source: Front Plant Sci. 2015 May 5;6:298. doi: 10.3389/fpls.2015.00298 (PMC4419667; doi:10.3389/fpls.2015.00298)
Supplement: Supplementary file 8 [file Table8.DOC]

**Table S8︱Maize proteins corresponding to rice proteins in network of protein interaction under heat.**

| **Maize query sequence** | **Rice query sequence** | **STRING protein** | **Identity** | **Bitscore** |
| --- | --- | --- | --- | --- |
| PIP27 | 4330049 | Aquaporin protein, putative, expressed; | 81% | 461 |
| NIP22 | 4340558 | Aquaporin protein, putative, expressed; | 86% | 518 |
| B4F7W7 | 4346661 | MYB-like DNA-binding domain containing protein, putative, expressed | 85% | 496 |
| B4F8M5 | 4346468 | Cytoskeletal protein, putative, expressed | 78% | 801 |
| B4F976 | 4332363 | HSP20/alpha crystallin family protein, putative | 81% | 246 |
| B4FAA8 | 4334896 | OsRhmbd10 - Putative Rhomboid homologue, expressed | 87% | 520 |
| B4FAB7 | 4327739 | CBS domain-containing protein, putative, expressed | 82% | 637 |
| B4FAU8 | 4349128 | Expressed protein | 86% | 795 |
| B4FBM8 | 4348719 | RNA recognition motif containing protein, putative, expressed | 80% | 475 |
| B4FC96 | 4343269 | Zinc finger family protein, putative, expressed | 76% | 376 |
| B4FGQ4 | 4326523 | Protein kinase APK1B, chloroplast precursor, putative, expressed | 90% | 571 |
| B4FKM0 | 4349004 | Oxidoreductase, short chain dehydrogenase/reductase family domain containing family, expressed; | 84% | 671 |
| B4FN85 | 4340496 | Mediator of RNA polymerase II transcription subunit 6, putative, expressed | 88% | 407 |
| B4FQ49 | 4326774 | Expressed protein | 89% | 329 |
| B4FQK5 | 4343757 | Eukaryotic peptide chain release factor subunit 1-1, putative, expressed | 96% | 876 |
| B4FQU2 | 4348536 | Initiation factor 2 subunit family domain containing protein, expressed | 94% | 784 |
| B4FRF2 | LOC_Os01g31690.1 | Oxygen-evolving enhancer protein 1, chloroplast precursor, putative, expressed | 93% | 444 |
| B4FXH5 | 4341864 | Surp module family protein, putative, expressed | 77% | 374 |
| B4FY17 | 4352524 | Phospholipase C, putative, expressed | 81% | 796 |
| B4FY62 | 4342409 | Zinc ion binding protein, putative, expressed | 80% | 736 |
| B4G250 | 4332363 | HSP20/alpha crystallin family protein, putative | 82% | 253 |
| B6SRN0 | 4334009 | Transposon protein, putative, unclassified, expressed | 86% | 1301 |
| B6SU00 | 4332355 | OsWLIM2 - LIM domain protein, putative actin-binding protein and transcription factor, expressed | 92% | 342 |
| B6SWV8 | 4344836 | CRS2-associated factor 2, mitochondrial precursor, putative, expressed; | 78% | 531 |
| B6SZ69 | 4332413 | DnaK family protein, putative, expressed | 94% | 1266 |
| B6T1H0 | 4333016 | 40S ribosomal protein S6, putative, expressed | 96% | 476 |
| B6T649 | 4330786 | Heat shock 22 kDa protein, mitochondrial precursor, putative, expressed | 76% | 298 |
| B6T8V1 | 4349880 | Protein of unknown function domain containing protein, expressed | 86% | 295 |
| B6TB14 | 4337644 | Splicing factor, arginine/serine-rich 7, putative, expressed | 77% | 188 |
| B6TH05 | 4349227 | Ethylene-responsive element-binding protein, putative, expressed | 85% | 419 |
| B6TJW1 | 4330410 | Expressed protein | 79% | 246 |
| B6TNC1 | 4336547 | Placental protein 11 precursor, putative, expressed | 77% | 706 |
| B6TWG6 | 4345944 | COP9 signalosome complex subunit 6a, putative, expressed | 89% | 600 |
| B6TWH2 | 4338911 | Soluble inorganic pyrophosphatase, putative, expressed | 89% | 381 |
| B6U4K3 | 4348376 | Vacuolar-sorting receptor precursor, putative, expressed | 85% | 1070 |
| B6U787 | 4336590 | Amino acid kinase, putative, expressed | 86% | 552 |
| B6U8P0 | 4334641 | Phosphoesterase family protein, putative, expressed | 95% | 1030 |
| B7ZYR5 | 4327723 | Inactive receptor kinase At2g26730 precursor, putative, expressed | 81% | 942 |
| B7ZZ27 | 4352301 | WRKY DNA-binding domain containing protein, expressed | 83% | 631 |
| B8A1A | 4325407 | Phosphoesterase family protein, putative, expressed | 80% | 837 |
| C0HE50 | 4341836 | Zinc finger, C3HC4 type, domain containing protein, expressed | 80% | 790 |
| C0HH76 | 4328603 | Lipoxygenase, putative, expressed; | 76% | 411 |
| C0HIM6 | 4329937 | Non-receptor tyrosine kinase spore lysis A, putative, expressed | 82% | 562 |
| C0P2N6 | 4339491 | Chaperone protein dnaJ 10, putative, expressed | 82% | 270 |
| C0P3W9 | 4332293 | Phosphoenolpyruvate carboxykinase, putative, expressed | 85% | 1129 |
| C0P4D8 | 4340644 | Dynamin, putative, expressed | 85% | 1474 |
| C0P9L7 | 4341497 | Copine-1, putative, expressed | 86% | 448 |
| C0PBP2 | 4348225 | Transcription initiation factor IIF, alpha subunit domain containing protein, expressed | 88% | 897 |
| C0PCR0 | 4346154 | Zinc finger DHHC domain-containing protein, putative, expressed | 85% | 469 |
| C0PD30 | 4349897 | Fructose-bisphospate aldolase isozyme, putative, expressed | 96% | 674 |
| C0PD66 | 4333506 | Expressed protein | 77% | 493 |
| C0PDN0 | 4324621 | Trehalose synthase, putative, expressed | 85% | 1548 |
| C0PE44 | 4335652 | Retrotransposon protein, putative, unclassified, expressed | 76% | 271 |
| C0PHB5 | 4330585 | RNA methyltransferase domain-containing protein 2, putative, expressed | 78% | 475 |
| C0PLZ2 | 4326444 | Peptide transporter PTR2, putative, expressed | 77% | 798 |
| C4J1T9 | 4351956 | Prefoldin, putative, expressed | 83% | 994 |
| C4J4G0 | 4325550 | Serine/threonine protein kinase, putative, expressed | 86% | 802 |
| C4J9J4 | 4332959 | KH domain-containing protein, putative, expressed | 83% | 828 |
| C4JBR4 | 4328119 | Glycine-rich protein 2, putative, expressed | 77% | 228 |
| E3UJZ2 | 4336442 | Transposon protein, putative, unclassified, expressed; | 88% | 1287 |
| F1DJV0 | 4327123 | Transcription factor HY5, putative, expressed | 77% | 183 |
| K7U162 | 4344699 | ELF7, putative, expressed | 85% | 792 |
| K7U573 | OsJ_21434 | PSP domain containing protein, expressed | 80% | 369 |
| K7U5U1 | 4329484 | DUF803 domain containing, putative, expressed | 81% | 289 |
| K7U5V4 | 4332722 | RNA-binding zinc finger protein, putative, expressed | 82% | 151 |
| K7UKJ5 | 4330968 | RNA recognition motif containing protein, expressed | 85% | 186 |
| K7UTP6 | 4345795 | Nitrate reductase, putative, expressed; | 90% | 1693 |
| K7V0H0 | 4330971 | Trehalose-6-phosphate synthase, putative, expressed | 88% | 1498 |
| K7V4D9 | 4339037 | HECT-domain domain containing protein, expressed | 76% | 544 |
| K7V5E8 | 4337834 | TOC159, putative, expressed | 94% | 1477 |
| K7V8I9 | LOC_Os01g55880.1 | Hemimethylated DNA binding domain containing protein | 81% | 541 |
| K7VBI0 | 4326743 | AAA-type ATPase family protein, putative, expressed | 85% | 1382 |
| K7VEK3 | 4340166 | Peptidase, T1 family, putative, expressed; | 78% | 437 |
| K7W2Z7 | 4331518 | Protein kinase family protein, putative, expressed | 83% | 728 |
| Q41729 | LOC_Os01g45274.1 | Carbonic anhydrase, chloroplast precursor, putative, expressed | 80% | 331 |
| Q8W149 | 4335542 | MYB family transcription factor, putative, expressed | 93% | 1326 |
| Q9LLI8 | 4337958 | CESA1 - cellulose synthase, expressed; | 96% | 2088 |
