# Supplementary material for: Phosphoproteomic analysis of the response of maize leaves to drought, heat and their combination stress
Source: Front Plant Sci. 2015 May 5;6:298. doi: 10.3389/fpls.2015.00298 (PMC4419667; doi:10.3389/fpls.2015.00298)
Supplement: Supplementary file 9 [file Table9.DOC]

**Table S9︱Maize proteins corresponding to rice proteins in network of protein interaction under combined stress.**

| **Maize query sequence** | **Rice query sequence** | **STRING protein** | **Identity** | **Bitscore** |
| --- | --- | --- | --- | --- |
| 14331 | 4336066 | 14-3-3 protein, putative, expressed | 98% | 516 |
| PIP27 | 4330049 | Aquaporin protein, putative, expressed; | 81% | 461 |
| NIP22 | 4340558 | Aquaporin protein, putative, expressed; | 86% | 518 |
| B4F7W7 | 4346661 | MYB-like DNA-binding domain containing protein, putative, expressed | 85% | 496 |
| B4F8R0 | 4343629 | Protein phosphatase 2C, putative, expressed | 87% | 746 |
| B4F976 | 4332363 | HSP20/alpha crystallin family protein, putative | 81% | 246 |
| B4FAA8 | 4334896 | OsRhmbd10 - Putative Rhomboid homologue, expressed | 87% | 520 |
| B4FAB7 | 4327739 | CBS domain-containing protein, putative, expressed | 82% | 637 |
| B4FAU8 | 4349128 | Expressed protein | 86% | 795 |
| B4FGQ4 | 4326523 | Protein kinase APK1B, chloroplast precursor, putative, expressed | 90% | 571 |
| B4FKM0 | 4349004 | Oxidoreductase, short chain dehydrogenase/reductase family domain containing family, expressed; | 84% | 671 |
| B4FQ49 | 4326774 | Expressed protein | 89% | 329 |
| B4FQK5 | 4343757 | Eukaryotic peptide chain release factor subunit 1-1, putative, expressed | 96% | 876 |
| B4FQU2 | 4348536 | Initiation factor 2 subunit family domain containing protein, expressed | 94% | 784 |
| B4FY17 | 4352524 | Phospholipase C, putative, expressed | 81% | 796 |
| B4FY62 | 4342409 | Zinc ion binding protein, putative, expressed | 80% | 736 |
| B4G250 | 4332363 | HSP20/alpha crystallin family protein, putative | 82% | 253 |
| B6SJ15 | 4330597 | AGAP000425-PA, putative, expressed | 81% | 206 |
| B6SKI1 | 4346326 | Photosystem I reaction center subunit II, chloroplast precursor, putative, expressed | 81% | 319 |
| B6SRN0 | 4334009 | Transposon protein, putative, unclassified, expressed | 86% | 1301 |
| B6SS20 | 4335426 | AGC_PVPK_like_kin82y.10 - ACG kinases include homologs to PKA, PKG and PKC, expressed | 77% | 1375 |
| B6SU00 | 4332355 | OsWLIM2 - LIM domain protein, putative actin-binding protein and transcription factor, expressed | 92% | 342 |
| B6SWV8 | 4344836 | CRS2-associated factor 2, mitochondrial precursor, putative, expressed; May be involved for the splicing of group IIB introns in mitochondrions (By similarity) | 78% | 531 |
| B6T1H0 | 4333016 | 40S ribosomal protein S6, putative, expressed | 96% | 476 |
| B6T890 | LOC_Os01g03500.1 | Expressed protein | 84% | 481 |
| B6T8V1 | 4349880 | Protein of unknown function domain containing protein, expressed | 86% | 295 |
| B6TB14 | 4337644 | Splicing factor, arginine/serine-rich 7, putative, expressed | 77% | 188 |
| B6TH05 | 4349227 | Ethylene-responsive element-binding protein, putative, expressed | 85% | 419 |
| B6TVL4 | 4330608 | Arsenate reductase, putative, expressed | 78% | 505 |
| B6TWH2 | 4338911 | Soluble inorganic pyrophosphatase, putative, expressed | 89% | 381 |
| B6TY90 | 4347295 | Gibberellin receptor GID1L2, putative, expressed | 78% | 538 |
| B6TZS3 | 4339909 | Expressed protein | 90% | 1012 |
| B6U4K3 | 4348376 | Vacuolar-sorting receptor precursor, putative, expressed | 85% | 1070 |
| B6U899 | 4339168 | Histone-lysine N-methyltransferase, H3 lysine-9 specific SUVH1, putative, expressed | 83% | 1179 |
| B6U8P0 | 4334641 | Phosphoesterase family protein, putative, expressed | 95% | 1030 |
| B7ZYP6 | 4338750 | Pyruvate, phosphate dikinase, chloroplast precursor, putative, expressed; Formation of phosphoenolpyruvate. The cytoplasmic isoform supports the biosynthetic processes in the nascent endosperm and provides an efficient mechanism for glycolytic ATP synthesis in oxygen depleted tissues. May be involved in regulating the flux of carbon into starch and fatty acids of seeds and in the remobilization of nitrogen reserves in senescing leaves | 86% | 1670 |
| B8A1A6 | 4325407 | Phosphoesterase family protein, putative, expressed | 80% | 837 |
| B8A326 | 4337257 | Plasma membrane ATPase, putative, expressed; The plasma membrane ATPase of plants and fungi is a hydrogen ion pump. The proton gradient it generates drives the active transport of nutrients by H(+)-symport. The resulting external acidification and/or internal alkinization may mediate growth responses (By similarity) | 96% | 1826 |
| C0HF02 | 4343583 | Chlorophyll A-B binding protein, putative, expressed | 86% | 264 |
| C0HH76 | 4328603 | Lipoxygenase, putative, expressed; Plant lipoxygenase may be involved in a number of diverse aspects of plant physiology including growth and development, pest resistance, and senescence or responses to wounding (By similarity) | 76% | 411 |
| C0P2N6 | 4339491 | Chaperone protein dnaJ 10, putative, expressed | 82% | 270 |
| C0P3W9 | 4332293 | Phosphoenolpyruvate carboxykinase, putative, expressed | 85% | 1129 |
| C0P4D8 | 4340644 | Dynamin, putative, expressed | 85% | 1474 |
| C0P8E4 | 4352419 | Plus-3 domain containing protein, expressed | 77% | 754 |
| C0P9L7 | 4341497 | Copine-1, putative, expressed | 86% | 448 |
| C0PD30 | 4349897 | Fructose-bisphospate aldolase isozyme, putative, expressed | 96% | 674 |
| C0PEW7 | 4327977 | Transmembrane amino acid transporter protein, putative, expressed | 83% | 849 |
| C0PHB5 | 4330585 | RNA methyltransferase domain-containing protein 2, putative, expressed | 78% | 475 |
| C0PLZ2 | 4326444 | Peptide transporter PTR2, putative, expressed | 77% | 798 |
| C0PM56 | 4333342 | Chloroplast post-illumination chlorophyll fluorescence increase protein, putative, expressed | 85% | 302 |
| C0PNT1 | 4352868 | RNA recognition motif containing protein, expressed | 96% | 57 |
| C4J1A8 | 4332374 | Protein phosphatase 2C, putative, expressed | 75% | 503 |
| C4J9J4 | 4332959 | KH domain-containing protein, putative, expressed | 83% | 828 |
| C4JBR4 | 4328119 | Glycine-rich protein 2, putative, expressed | 77% | 228 |
| E9NQE1 | 4328859 | Phosphoenolpyruvate carboxylase, putative, expressed | 82% | 1607 |
| F1DJV0 | 4327123 | Transcription factor HY5, putative, expressed | 77% | 183 |
| K7TLV1 | 4336331 | Polyadenylate-binding protein, putative, expressed | 84% | 326 |
| K7TUM2 | 4336300 | Eukaryotic initiation factor iso-4F subunit p82-34, putative, expressed | 79% | 842 |
| K7U162 | 4344699 | ELF7, putative, expressed | 85% | 792 |
| K7U573 | OsJ_21434 | PSP domain containing protein, expressed | 80% | 369 |
| K7U5V4 | 4332722 | RNA-binding zinc finger protein, putative, expressed | 82% | 151 |
| K7UAY1 | 4343083 | Protein kinase domain containing protein, expressed | 76% | 1805 |
| K7UBY5 | 4348127 | RNA recognition motif containing protein, expressed | 77% | 594 |
| K7UKJ5 | 4330968 | RNA recognition motif containing protein, expressed | 85% | 186 |
| K7V1I2 | LOC_Os01g72890.1 | Transposon protein, putative, CACTA, En/Spm sub-class, expressed | 86% | 322 |
| K7V792 | OsJ_05362 | Splicing factor 3B subunit 1, putative, expressed | 95% | 2380 |
| Q41729 | LOC_Os01g45274.1 | Carbonic anhydrase, chloroplast precursor, putative, expressed | 80% | 331 |
| Q5QJA2 | 4350844 | PAP fibrillin family domain containing protein, expressed | 85% | 352 |
| Q8W149 | 4335542 | MYB family transcription factor, putative, expressed | 93% | 1326 |
| Q9LLI8 | 4337958 | CESA1 - cellulose synthase, expressed; Probable catalytic subunit of cellulose synthase terminal complexes ('rosettes'), required for beta-1,4-glucan microfibril crystallization, a major mechanism of the cell wall formation (By similarity) | 96% | 2088 |
